# Post-marketing safety signal profile of mavacamten in the FDA adverse event reporting system (FAERS): a disproportionality analysis

**DOI:** 10.3389/fphar.2026.1906371

**Published:** 2026-09-09

**Authors:** Juan Wu, Chi Chen, Tao Zheng

**Affiliations:** 1 Department of Cardiology, The First Affiliated Hospital of Guizhou University of Traditional Chinese Medicine, Guiyang, Guizhou, China; 2 Department of Preventive Medicine, School of Public Health, Guizhou University of Traditional Chinese Medicine, Guiyang, Guizhou, China

**Keywords:** camzyos, cardiac myosin inhibitor, disproportionality analysis, FAERS, mavacamten, obstructive hypertrophic cardiomyopathy, pharmacovigilance

## Abstract

**Background:**

Mavacamten is a first-in-class cardiac myosin inhibitor approved for symptomatic obstructive hypertrophic cardiomyopathy; however, its risk of reduced left ventricular ejection fraction and systolic heart failure warrants post-marketing safety evaluation.

**Methods:**

FAERS quarterly open data from 2022 Q2 to 2025 Q2 (13 quarters) were retrospectively analyzed. After global deduplication, mavacamten reports were identified using “MAVACAMTEN” or “CAMZYOS” as primary or secondary suspect drugs. Disproportionality was assessed by the reporting odds ratio (ROR), proportional reporting ratio (PRR), information component (IC), and empirical Bayes geometric mean (EBGM). A positive primary signal required at least 3 reports, a lower bound of the ROR 95% confidence interval (CI) greater than 1, and IC_025_ greater than 0; PRR and EBGM served as consistency measures only. Sensitivity analyses included count-threshold analyses and an exploratory covariate-adjusted ROR (aROR) analysis.

**Results:**

Among 4,514,490 deduplicated FAERS cases, 2,933 mavacamten-associated reports were identified, containing 955 unique preferred terms (PTs). Eighty PTs met the primary signal criterion (68 clinical, 9 administrative/product-use, 3 ambiguous); 45 were concordant across all four algorithms. Ejection fraction decreased was a prominent labeled cardiac safety signal (a = 99; ROR 47.08, 95% CI 38.42–57.69; aROR 54.74, 95% CI 44.53–67.30), together with atrial fibrillation (a = 205; ROR 17.63, 95% CI 15.28–20.33; aROR 14.52, 95% CI 12.56–16.78) and cardiac failure (a = 57; ROR 5.55, 95% CI 4.27–7.22; aROR 5.10, 95% CI 3.92–6.64). In the exploratory adjusted analysis, 79 of 80 signals remained significant; only cataract did not (aROR 1.54, 95% CI 0.96–2.48; p = 0.076).

**Conclusion:**

Mavacamten was associated with expected cardiac safety signals and additional exploratory signals, supporting continued surveillance and careful cardiac monitoring. These hypothesis-generating findings require confirmation in independent data sources.

## 1 Introduction

Hypertrophic cardiomyopathy (HCM) is the most common inherited cardiomyopathy and is characterized by unexplained left ventricular hypertrophy, myocyte disarray, dynamic left ventricular outflow tract (LVOT) obstruction, diastolic dysfunction, and an increased risk of arrhythmia and heart failure symptoms (Ommen et al., 2020; Elliott et al., 2014). In patients with obstructive HCM, hypercontractility and dynamic LVOT obstruction contribute substantially to exertional dyspnea, chest discomfort, syncope, reduced functional capacity, and impaired quality of life (Ommen et al., 2020; Elliott et al., 2014). Contemporary guidelines recommend individualized treatment strategies, including negative inotropic pharmacotherapy, septal reduction therapy in selected patients, and longitudinal surveillance for disease progression and complications (Ommen et al., 2020; Elliott et al., 2014).

Mavacamten is a first-in-class selective cardiac myosin inhibitor developed to target the underlying sarcomeric hypercontractility of obstructive HCM (Green et al., 2016; Olivotto et al., 2020). By reducing excessive actin–myosin cross-bridge formation, mavacamten decreases myocardial contractility and can reduce LVOT gradients. In the pivotal phase 3 EXPLORER-HCM trial, mavacamten improved exercise capacity, symptoms, NYHA functional class, and LVOT obstruction among patients with symptomatic obstructive HCM (Olivotto et al., 2020). Additional evidence from VALOR-HCM showed that mavacamten reduced eligibility for septal reduction therapy in patients with severe symptomatic obstructive HCM who had been referred for invasive treatment (Desai et al., 2022; Desai et al., 2023). Based on these data, mavacamten was approved by the U.S. Food and Drug Administration in 2022 for adults with symptomatic NYHA class II–III obstructive HCM (U.S. Food and Drug Administration, 2022).

Despite its therapeutic benefits, mavacamten has an important safety profile that requires post-marketing surveillance. Because mavacamten reduces left ventricular ejection fraction (LVEF), excessive pharmacologic exposure or patient susceptibility may lead to systolic dysfunction and heart failure. Accordingly, the U.S. prescribing information includes a boxed warning for the risk of heart failure and recommends echocardiographic assessment of LVEF before and during treatment (U.S. Food and Drug Administration, 2022). Clinical trials provide controlled evidence on efficacy and common adverse events, but their sample sizes, eligibility criteria, and follow-up durations may limit the detection of rare, delayed, or real-world safety signals. Spontaneous reporting systems such as the FDA Adverse Event Reporting System (FAERS) therefore provide a complementary resource for post-marketing pharmacovigilance, particularly for newly approved therapies (U.S. Food and Drug Administration, 2026; Sakaeda et al., 2013).

Disproportionality analysis of spontaneous reporting data is widely used to identify drug–event combinations reported more frequently than expected relative to a background reporting database. Commonly applied measures include the reporting odds ratio (ROR), proportional reporting ratio (PRR), information component (IC), and empirical Bayes geometric mean (EBGM) (E et al., 2001; van Puijenbroek et al., 2002; Bate et al., 1998; DuMouchel, 1999). These approaches are hypothesis-generating and cannot establish incidence, absolute risk, or causality, but they can help detect potential safety concerns that warrant further clinical or epidemiologic evaluation (U.S. Food and Drug Administration, 2026; Sakaeda et al., 2013; E et al., 2001; van Puijenbroek et al., 2002; Bate et al., 1998; DuMouchel, 1999).

An earlier FAERS-based disproportionality analysis of mavacamten was published by Yukselen et al. (2024), covering reports accrued from January 2022 through September 2023. To position the present study relative to that report without mixing analysis windows, we re-derived estimates restricted to the aligned post-approval period available in the present database (2022 Q2–2023 Q3); this period is described as aligned rather than identical to the published calendar window because accrual in the present database begins with the post-approval quarters. Over this aligned period, the present dataset contained 1,003 mavacamten cases, versus 1,004 unique cases reported by Yukselen et al., and the corresponding estimates were concordant in direction and broadly similar in magnitude: ejection fraction decreased, ROR 29.09 (95% CI 19.01–44.51) in the present same-window analysis versus 33.60 (21.79–51.82) in Yukselen et al.; atrial fibrillation, 15.89 (12.32–20.48) versus 16.63 (12.72–21.75); and cardiac failure, 9.48 (6.70–13.42) versus 9.39 (6.49–13.60) (Supplementary Table S7). The present study extends that earlier work by analyzing a longer post-approval period of 13 quarters (2,933 mavacamten reports, approximately 2.9-fold the same-window case count), applying a prespecified three-part primary signal criterion, count-threshold sensitivity analyses, and an exploratory covariate-adjusted analysis.

Given the recent approval of mavacamten and the need for real-world safety evaluation, this study analyzed FAERS reports from 2022 Q2 through 2025 Q2 to characterize adverse event reporting patterns associated with mavacamten. We aimed to identify disproportionality signals, evaluate clinically relevant cardiovascular and non-cardiovascular events, and assess signal robustness using multi-algorithm consistency, count-threshold sensitivity analyses, and covariate-adjusted sensitivity analyses.

## 2 Methods

### 2.1 Data source and study design

**FIGURE 1:**
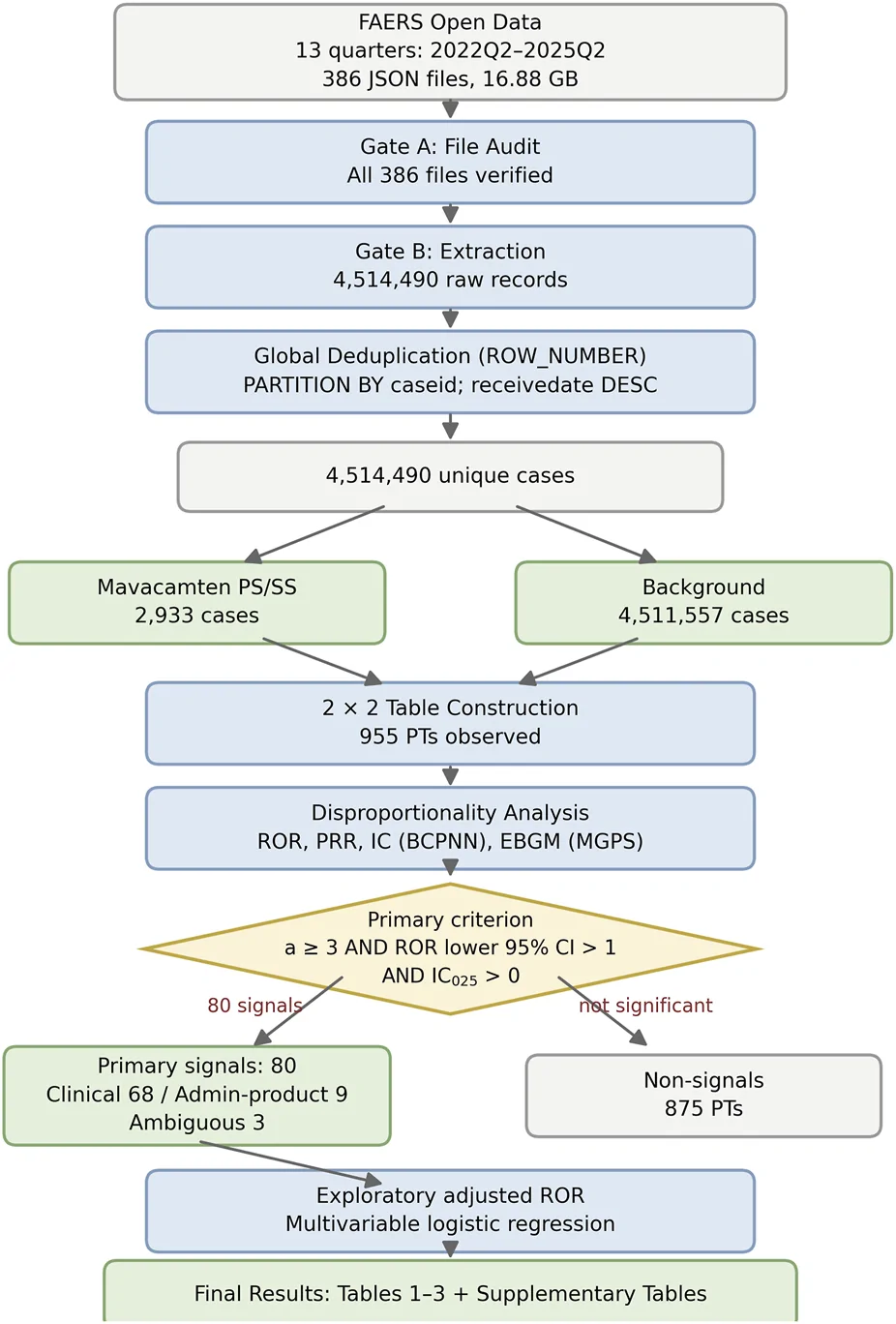
Data extraction and study flow diagram. Flow diagram showing the identification of mavacamten-associated reports from the FDA Adverse Event Reporting System (FAERS) quarterly open data files, 2022 Q2 to 2025 Q2 (13 quarters; 386 quarterly data files). After file audit and extraction, 4,514,490 unique cases were retained following global deduplication (ROW_NUMBER() OVER (PARTITION BY caseid ORDER BY receivedate DESC, version DESC)). Mavacamten reports were identified using the drug names “MAVACAMTEN” and “CAMZYOS” with primary suspect or secondary suspect role codes (2,933 cases); the remaining 4,511,557 reports formed the background. A total of 955 unique preferred terms were evaluated. Applying the primary criterion (a ≥ 3, ROR 95% CI lower bound > 1, and IC_025_ > 0) yielded 80 primary signals (68 clinical, 9 administrative/product-use, 3 ambiguous) and 875 non-signals; 45 signals were concordant across all four algorithms. Exploratory adjusted ROR models were fitted for all 80 signals. ROR, reporting odds ratio; PRR, proportional reporting ratio; IC, information component; EBGM, empirical Bayes geometric mean; aROR, adjusted reporting odds ratio.

We conducted a retrospective pharmacovigilance study using the FDA Adverse Event Reporting System (FAERS) quarterly open data files publicly released by the U.S. Food and Drug Administration. The study period extended from 2022 Q2, the quarter in which mavacamten received FDA approval, through 2025 Q2, covering 13 quarters of quarterly data files. FAERS is a spontaneous reporting database that includes adverse event reports submitted by healthcare professionals, consumers, manufacturers, and other reporters from the United States and other countries. Because FAERS is a publicly available, de-identified database, institutional review board approval and informed consent were not required. The overall data extraction and analytic workflow is summarized in Figure 1. Across the study window, 4,514,490 unique deduplicated FAERS cases were available for analysis, of which 2,933 were mavacamten-associated reports (primary or secondary suspect); the remaining 4,511,557 reports constituted the comparative background population.

### 2.2 Identification of mavacamten reports and deduplication

Mavacamten reports were identified using both the generic and brand names, “MAVACAMTEN” and “CAMZYOS.” Drug records were screened using the available drug-identification fields, including medicinal product name and active substance name. Reports in which mavacamten was coded as either primary suspect (PS) or secondary suspect (SS) were included.

Deduplication was performed globally across the full extraction using the FDA-recommended approach, implemented as ROW_NUMBER() OVER (PARTITION BY caseid ORDER BY receivedate DESC, version DESC): for each unique CASEID, the record with the most recent receipt date was retained, and when multiple records shared the same receipt date, the record with the highest version number was selected. This procedure yielded 2,933 unique mavacamten-associated reports (PS/SS). For comparative disproportionality analyses, all other deduplicated FAERS reports during the same study period (n = 4,511,557) were used as the background population.

### 2.3 Data elements and descriptive analysis

For each mavacamten-associated report, we extracted patient demographics (age category and sex), reporter type, reporting country, report year, seriousness, death outcome, drug role, and adverse events coded as preferred terms (PTs). Age completeness and sex completeness were assessed before analysis. Descriptive statistics were used to summarize the baseline characteristics of the mavacamten reports. Categorical variables are reported as counts and percentages, with unknown or missing values retained as explicit categories.

### 2.4 Disproportionality analysis

Disproportionality was assessed at the mavacamten–PT level. For each PT, a 2 × 2 contingency table was constructed comparing the reporting frequency of the target PT among mavacamten-associated reports with the reporting frequency of the same PT among all other FAERS reports in the study period. The four cells were defined as follows: a, the number of mavacamten reports containing the target PT; b, the number of mavacamten reports containing other PTs; c, the number of non-mavacamten reports containing the target PT; and d, the number of non-mavacamten reports containing other PTs.

The primary disproportionality metric was the reporting odds ratio (ROR), calculated as (a × d)/(b × c). Ninety-five percent confidence intervals (CIs) for the ROR were derived using the log-normal approximation. The proportional reporting ratio (PRR), the Bayesian confidence propagation neural network information component (IC), and the empirical Bayes geometric mean (EBGM, Multi-Gamma Poisson Shrinker) were also computed; however, PRR and EBGM were used exclusively as consistency and robustness measures and were not incorporated into the primary signal definition. The Haldane correction (0.5 added to all cells) was applied when required for sparse contingency tables with a zero cell.

A positive primary disproportionality signal was defined using a prespecified three-part criterion: at least three mavacamten reports for the PT (a ≥ 3), a lower bound of the ROR 95% CI greater than 1.0, and an IC lower credibility bound (IC_025_) greater than 0. This combined frequentist–Bayesian criterion was used to improve robustness and reduce the likelihood of false-positive findings. As a robustness stratification only (not part of the primary criterion), four-algorithm concordance was defined as a ≥ 3, ROR 95% CI lower bound > 1, PRR ≥ 2 with χ^2^ ≥ 4, IC_025_ > 0, and EB05 > 2. In addition, count-threshold sensitivity analyses were performed by re-applying the primary criterion at minimum report counts of a ≥ 5 and a ≥ 10. PTs meeting the primary criterion were summarized by System Organ Class (SOC) and were further classified as clinical adverse events, administrative/product-use terms, or ambiguous terms; administrative/product-use PTs are reported separately from clinical adverse events.

### 2.5 Exploratory sensitivity analysis using adjusted reporting odds ratios

To evaluate whether disproportionality signals were robust to adjustment for basic report-level covariates, multivariable logistic regression was performed for each primary-signal PT as an exploratory analysis. The dependent variable was the presence of the target PT, and the primary independent variable was mavacamten exposure. Models were adjusted for age category (<18, 18–44, 45–64, 65–79, and ≥80 years, with unknown age retained as a separate category), sex (female/male/unknown), report year (continuous, centered at 2023), and healthcare-professional reporter status (FAERS reporter occupation codes 1, 2, 3, and 6). These covariates were selected *a priori* because they are the principal report-level variables consistently available in FAERS that may be associated with both reporting behavior and event mix; clinical covariates such as HCM severity are not captured in FAERS and therefore could not be included.

No case deletion was performed for missing covariates: unknown age and unknown sex were modeled as explicit categories rather than imputed or dropped. At the model level (database-wide), covariate missingness was 42.4% for age and 16.3% for sex. The exponentiated coefficient for mavacamten exposure was reported as the adjusted reporting odds ratio (aROR) with 95% CI. All 80 models converged (13 iterations each), with no evidence of separation, a condition number of the covariate correlation matrix of 6.9, and a maximum variance inflation factor of 2.0; all 80 models were classified as stable. Signals with an aROR 95% CI lower bound greater than 1.0 were considered consistent with the crude result after adjustment. Because of the substantial covariate missingness, the absence of HCM severity and other clinical variables, and the hypothesis-generating nature of the analysis, the aROR results are presented strictly as an exploratory adjusted analysis. Crude and adjusted estimates are presented in Table 3 and visualized for selected PTs in Figure 4. Per-model diagnostics are reported in Supplementary Table S6.

## 3 Results

### 3.1 Case identification and baseline characteristics

The FAERS quarterly data extraction identified 4,514,490 unique deduplicated cases during the 2022 Q2–2025 Q2 analysis window (13 quarters). Among these, 2,933 reports in which mavacamten was coded as primary or secondary suspect were included in the final analysis cohort, and 4,511,557 reports formed the background population (Figure 1). The mavacamten reports contained 955 unique PTs that were evaluated in the primary disproportionality analysis.

Baseline characteristics of the 2,933 mavacamten-associated reports are shown in Table 1. Most reports involved female patients (1,716, 58.5%), followed by male patients (793, 27.0%); sex was unknown in 424 reports (14.5%). The largest age group was 65–79 years (1,034, 35.3%), followed by 45–64 years (652, 22.2%), 80 years or older (311, 10.6%), 18–44 years (151, 5.1%), and younger than 18 years (29, 1.0%); age was unknown in 756 reports (25.8%).

**TABLE 1:** Baseline characteristics of mavacamten-associated reports in FAERS, 2022 Q2–2025 Q2 (N = 2,933).

Variable	Mavacamten reports (N = 2,933)	Background (N = 4,511,557)
Total mavacamten reports (PS + SS)	2,933	4,511,557
Sex: Female	1,716 (58.5%)	
Sex: Male	793 (27.0%)	
Sex: Unknown/missing	424 (14.5%)	
Age: <18	29 (1.0%)	
Age: 18–44	151 (5.1%)	
Age: 45–64	652 (22.2%)	
Age: 65–79	1,034 (35.3%)	
Age: 80+	311 (10.6%)	
Age: Unknown	756 (25.8%)	
Reporter: HCP	1,349 (46.0%)	
Reporter: Consumer	0 (0.0%)	
Reporter: Other/Unknown	1,584 (54.0%)	
Country: US	2,661 (90.7%)	
Country: CA	61 (2.1%)	
Country: GB	38 (1.3%)	
Country: FR	33 (1.1%)	
Country: AU	29 (1.0%)	
Year: 2022	385 (13.1%)	
Year: 2023	810 (27.6%)	
Year: 2024	1,028 (35.0%)	
Year: 2025	710 (24.2%)	
Drug role: PS only	2,261 (77.1%)	
Drug role: includes SS	672 (22.9%)	
Serious reports	1,109 (37.8%)	
Death outcomes	71 (2.4%)	

Source file: table1_demographics_revised.csv. Values are n (%); percentages are calculated over all 2,933 mavacamten reports, with unknown/missing values retained as explicit categories. Background = all other deduplicated FAERS reports in the same window (N = 4,511,557); background subgroup breakdowns are not shown. PS, primary suspect; SS, secondary suspect; HCP, healthcare professional; FAERS = FDA, adverse event reporting system.

The United States accounted for most reports (2,661, 90.7%), followed by Canada (61, 2.1%), the United Kingdom (38, 1.3%), France (33, 1.1%), and Australia (29, 1.0%). Healthcare professionals submitted 1,349 reports (46.0%); no reports were coded as consumer-submitted, and 1,584 reports (54.0%) were from other or unknown reporter types. Report counts by year were 385 (13.1%) in 2022, 810 (27.6%) in 2023, 1,028 (35.0%) in 2024, and 710 (24.2%) in 2025; 2022 and 2025 represent partial calendar years within the 13-quarter window. Mavacamten was the sole primary suspect in 2,261 reports (77.1%), and 672 reports (22.9%) included a secondary-suspect designation. Serious reports accounted for 1,109 cases (37.8%), and death was reported as an outcome in 71 cases (2.4%).

### 3.2 Primary disproportionality signals

**FIGURE 2:**
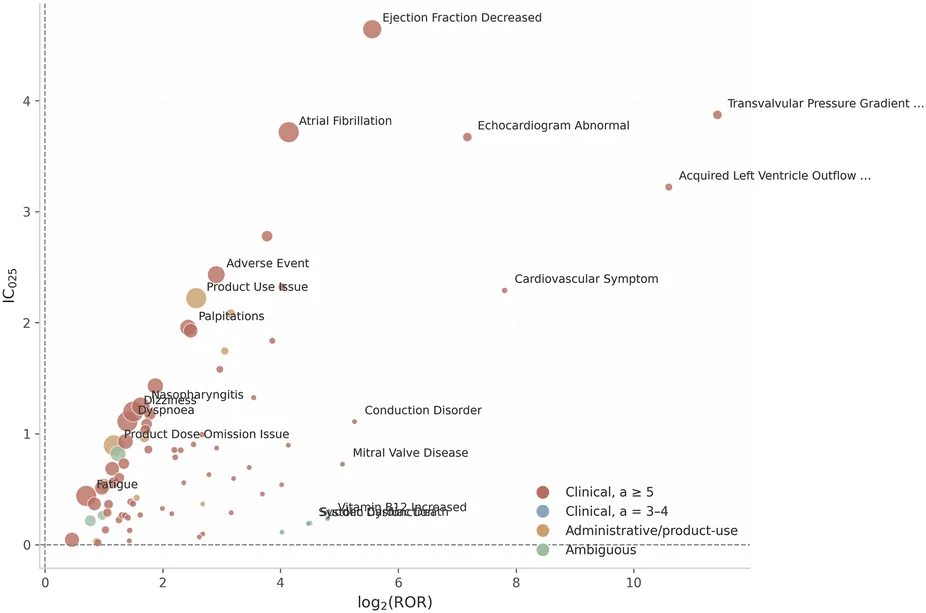
Volcano plot of the 80 primary mavacamten-associated disproportionality signals. Each point represents one preferred term meeting the primary signal criterion (a ≥ 3, ROR 95% CI lower bound > 1, and IC_025_ > 0). The x-axis shows the effect size on the log_2_ (ROR) scale, with a vertical reference line at log_2_ (ROR) = 0 (ROR = 1, no association). The y-axis shows IC_025_, the lower 95% credibility bound of the information component, with a horizontal reference line at IC_025_ = 0. Colors distinguish clinical signals with a ≥ 5 reports (red), clinical signals with a = 3–4 reports (blue), administrative/product-use PTs (orange, n = 9), and ambiguous PTs (teal, n = 3); 80 points are plotted in total (68 clinical). Selected PTs (top 10 by ROR and top 10 by report count) are labeled. ROR, reporting odds ratio; IC, information component; PT, preferred term.

**FIGURE 3:**
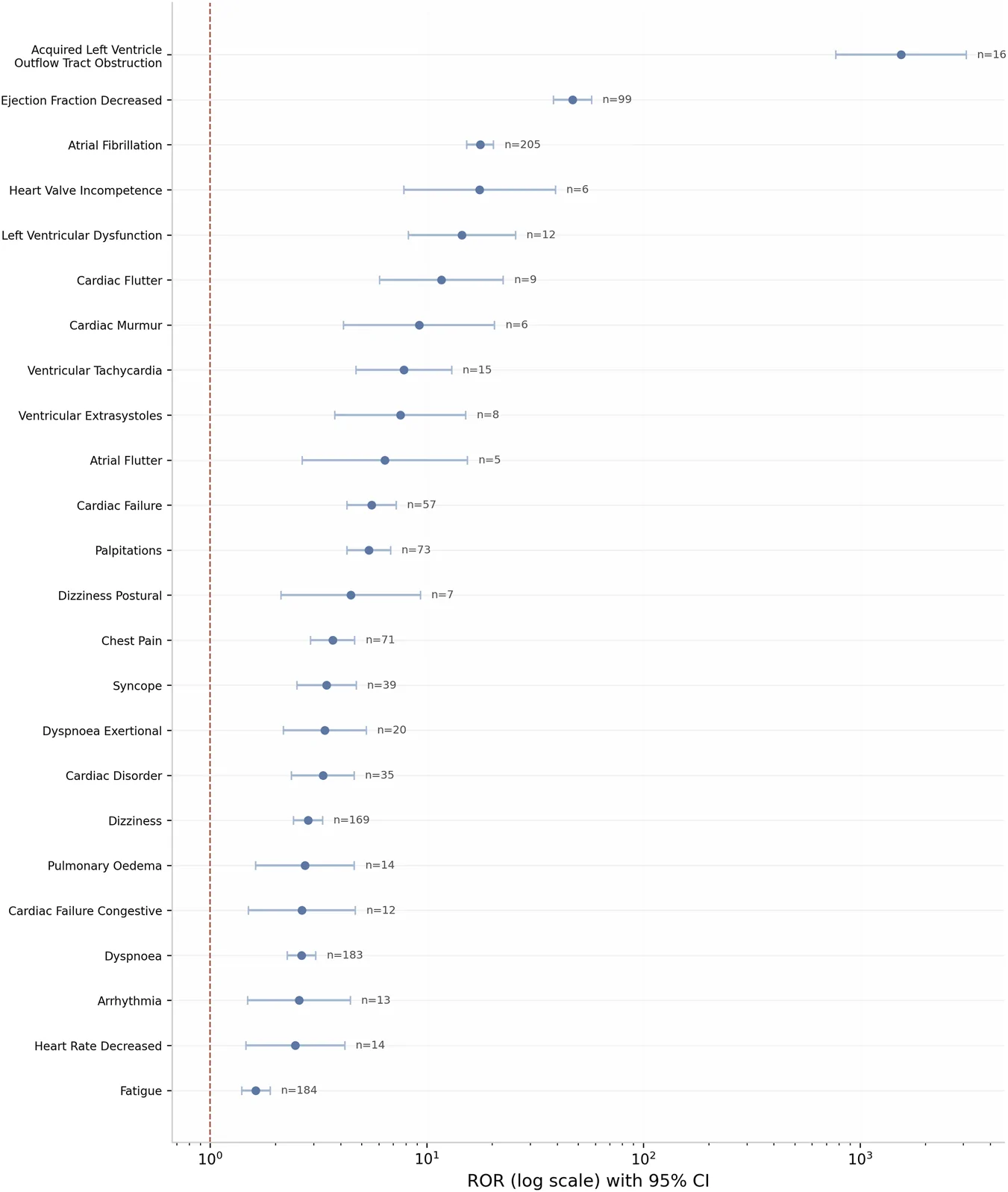
Forest plot of 24 prioritized clinical signals associated with mavacamten. Forest plot showing 24 clinical preferred terms with at least five reports that met the primary signal criterion, prioritized by biological plausibility (cardiac, neurological, and vascular events) rather than by ROR ranking alone. Points represent the ROR and horizontal lines the 95% confidence intervals on a logarithmic scale; the vertical reference line indicates ROR = 1.0. Estimates based on small report counts (e.g., atrial flutter, a = 5) have wide confidence intervals and are displayed for completeness rather than emphasis. ROR, reporting odds ratio; CI, confidence interval; PT, preferred term.

Among the 955 unique PTs observed in mavacamten-associated reports, 80 met the prespecified three-part primary criterion (a ≥ 3, ROR 95% CI lower bound > 1, and IC_025_ > 0): 72 were classified as clinical adverse events, 5 as administrative/product-use terms, and 3 as ambiguous terms. Forty-five signals were concordant across all four disproportionality algorithms (a robustness stratification, not part of the primary criterion). Count-threshold sensitivity analyses showed that the signal set was stable at higher minimum report counts: 76 signals (64 clinical) at a ≥ 5 and 58 signals (47 clinical) at a ≥ 10 (Supplementary Table S3). The 64 main clinical signals are summarized in Table 2, visualized in the volcano plot in Figure 2, and displayed for 24 biologically prioritized clinical signals in the forest plot in Figure 3; the complete 80-signal list is provided in Supplementary Table S1.

**TABLE 2:** Primary clinical disproportionality signals of mavacamten in FAERS (64 main clinical rows).

Preferred term (PT)	N (a)	ROR (95% CI)	IC (IC_025_)	EBGM (EB_05_)	Four-algorithm concordant	aROR (95% CI)†	p-value†
Atrial fibrillation	205	17.63 (15.28–20.33)	3.92 (3.72)	16.26 (14.47)	Yes	14.52 (12.56–16.78)	<0.001
Fatigue	184	1.62 (1.40–1.89)	0.66 (0.44)	1.47 (1.28)	No	1.60 (1.37–1.85)	<0.001
Dyspnoea	183	2.64 (2.27–3.07)	1.33 (1.11)	2.53 (2.24)	Yes	2.50 (2.15–2.90)	<0.001
Dizziness	169	2.83 (2.42–3.30)	1.42 (1.20)	2.71 (2.39)	Yes	2.71 (2.32–3.17)	<0.001
Ejection fraction decreased	99	47.08 (38.42–57.69)	4.94 (4.64)	43.86 (37.06)	Yes	54.74 (44.53–67.30)	<0.001
Nasopharyngitis	92	3.09 (2.51–3.81)	1.56 (1.25)	3.01 (2.52)	Yes	3.27 (2.65–4.02)	<0.001
Adverse event	85	7.51 (6.05–9.32)	2.76 (2.43)	7.25 (6.04)	Yes	7.04 (5.67–8.75)	<0.001
Palpitations	73	5.39 (4.27–6.80)	2.31 (1.96)	5.23 (4.30)	Yes	5.35 (4.24–6.76)	<0.001
Chest pain	71	3.67 (2.90–4.64)	1.79 (1.43)	3.57 (2.93)	Yes	3.69 (2.92–4.68)	<0.001
Urinary tract infection	65	2.58 (2.02–3.30)	1.30 (0.93)	2.52 (2.05)	Yes	2.25 (1.75–2.87)	<0.001
Cough	61	1.37 (1.07–1.77)	0.43 (0.04)	1.17 (1.00)	No	1.34 (1.04–1.73)	0.024
Peripheral swelling	57	2.21 (1.70–2.87)	1.08 (0.68)	2.13 (1.62)	No	2.02 (1.55–2.64)	<0.001
Cardiac failure	57	5.55 (4.27–7.22)	2.33 (1.93)	5.40 (4.32)	Yes	5.10 (3.92–6.64)	<0.001
Hypertension	56	1.95 (1.50–2.55)	0.92 (0.51)	1.74 (1.22)	No	1.96 (1.51–2.56)	<0.001
Hospitalisation	51	1.79 (1.36–2.36)	0.79 (0.37)	1.43 (1.10)	No	1.74 (1.32–2.30)	<0.001
Syncope	39	3.44 (2.51–4.72)	1.66 (1.18)	3.36 (2.56)	Yes	3.29 (2.40–4.52)	<0.001
Tooth disorder	35	13.66 (9.77–19.09)	3.30 (2.78)	13.19 (9.89)	Yes	12.59 (9.00–17.63)	<0.001
Chest discomfort	35	2.53 (1.81–3.53)	1.25 (0.73)	2.43 (1.72)	No	2.40 (1.71–3.37)	<0.001
Cardiac disorder	35	3.31 (2.37–4.62)	1.60 (1.09)	3.23 (2.43)	Yes	3.02 (2.16–4.22)	<0.001
Sinusitis	34	2.25 (1.60–3.15)	1.09 (0.56)	1.99 (1.20)	No	2.44 (1.74–3.42)	<0.001
Gastrooesophageal reflux disease	32	3.26 (2.30–4.62)	1.58 (1.03)	3.18 (2.35)	Yes	3.24 (2.29–4.60)	<0.001
Dyspepsia	30	2.40 (1.67–3.43)	1.16 (0.60)	2.16 (1.22)	No	2.32 (1.62–3.33)	<0.001
Arthritis	25	2.11 (1.43–3.14)	0.98 (0.36)	1.51 (1.03)	No	2.10 (1.41–3.11)	<0.001
Transvalvular pressure gradient increased	23	2742.94 (1388.14–5420.02)	4.52 (3.87)	676.21 (472.69)	Yes	2700.12 (1263.61–5769.68)	<0.001
Echocardiogram abnormal	23	144.36 (94.02–221.66)	4.32 (3.67)	121.77 (85.12)	Yes	181.43 (116.42–282.74)	<0.001
Rhinorrhoea	22	2.08 (1.37–3.17)	0.95 (0.29)	1.39 (0.99)	No	2.11 (1.38–3.21)	<0.001
Dyspnoea exertional	20	3.38 (2.18–5.25)	1.56 (0.86)	3.23 (2.14)	Yes	2.95 (1.90–4.58)	<0.001
Cataract	17	1.86 (1.16–3.00)	0.79 (0.02)	1.18 (0.92)	No	1.54 (0.96–2.48)	0.076
Blood pressure decreased	17	2.04 (1.26–3.28)	0.90 (0.13)	1.25 (0.94)	No	1.92 (1.19–3.09)	0.008
Mitral valve incompetence	17	16.32 (10.11–26.36)	3.09 (2.32)	15.53 (10.20)	Yes	16.41 (10.13–26.57)	<0.001
Acquired left ventricle outflow tract obstruction	16	1546.64 (772.73–3095.65)	4.01 (3.22)	505.96 (327.90)	Yes	2101.83 (973.25–4539.10)	<0.001
Ventricular tachycardia	15	7.83 (4.71–13.02)	2.40 (1.58)	7.50 (4.79)	Yes	8.03 (4.82–13.38)	<0.001
Heart rate decreased	14	2.47 (1.46–4.18)	1.12 (0.26)	1.49 (0.95)	No	2.64 (1.53–4.55)	<0.001
Pulmonary oedema	14	2.74 (1.62–4.63)	1.24 (0.39)	1.83 (0.99)	No	2.43 (1.44–4.11)	<0.001
Fluid retention	14	2.39 (1.41–4.04)	1.08 (0.22)	1.41 (0.94)	No	2.23 (1.32–3.77)	0.003
Arrhythmia	13	2.57 (1.49–4.43)	1.15 (0.26)	1.53 (0.95)	No	2.50 (1.45–4.31)	0.001
Fungal infection	13	2.81 (1.63–4.85)	1.26 (0.37)	1.83 (0.98)	No	3.02 (1.75–5.21)	<0.001
Angina pectoris	12	4.59 (2.60–8.09)	1.78 (0.85)	4.27 (2.44)	Yes	4.47 (2.53–7.90)	<0.001
Cardiac failure congestive	12	2.65 (1.50–4.68)	1.17 (0.24)	1.54 (0.94)	No	2.31 (1.31–4.07)	0.004
Left ventricular dysfunction	12	14.53 (8.22–25.69)	2.77 (1.84)	13.68 (8.25)	Yes	15.35 (8.66–27.21)	<0.001
Respiratory tract congestion	11	4.94 (2.73–8.94)	1.83 (0.85)	4.58 (2.55)	Yes	4.46 (2.46–8.08)	<0.001
Gout	11	4.63 (2.56–8.38)	1.76 (0.79)	4.22 (2.18)	Yes	4.43 (2.45–8.03)	<0.001
Cardiovascular symptom	10	223.69 (115.13–434.61)	3.32 (2.29)	156.22 (89.38)	Yes	252.04 (126.46–502.31)	<0.001
Coronavirus infection	10	5.75 (3.09–10.71)	1.93 (0.90)	5.35 (2.98)	Yes	6.69 (3.59–12.48)	<0.001
Cardiomyopathy	10	6.35 (3.41–11.83)	2.02 (0.99)	5.95 (3.37)	Yes	6.47 (3.47–12.07)	<0.001
Diverticulitis	10	2.71 (1.46–5.04)	1.16 (0.13)	1.43 (0.91)	No	2.38 (1.28–4.44)	0.006
Blood pressure abnormal	10	3.07 (1.65–5.72)	1.30 (0.27)	1.77 (0.94)	No	2.76 (1.48–5.13)	0.001
Cardiac flutter	9	11.68 (6.05–22.52)	2.42 (1.32)	10.88 (6.02)	Yes	11.08 (5.73–21.43)	<0.001
Haematuria	9	2.70 (1.40–5.19)	1.13 (0.04)	1.35 (0.90)	No	2.39 (1.19–4.79)	0.014
Gallbladder disorder	8	5.12 (2.56–10.27)	1.73 (0.56)	4.15 (1.12)	No	4.91 (2.45–9.85)	<0.001
Tooth infection	8	3.98 (1.99–7.98)	1.49 (0.33)	2.49 (0.96)	No	4.33 (2.16–8.68)	<0.001
Ventricular extrasystoles	8	7.54 (3.76–15.11)	2.04 (0.87)	6.91 (3.60)	Yes	7.72 (3.85–15.51)	<0.001
Stress cardiomyopathy	7	6.89 (3.28–14.49)	1.89 (0.63)	5.96 (2.36)	Yes	5.09 (2.41–10.72)	<0.001
Dizziness postural	7	4.45 (2.12–9.35)	1.54 (0.28)	2.71 (0.96)	No	3.93 (1.87–8.27)	<0.001
Cardiac murmur	6	9.21 (4.12–20.56)	1.97 (0.60)	8.01 (3.48)	Yes	9.35 (4.18–20.93)	<0.001
Conduction disorder	6	38.21 (16.99–85.97)	2.48 (1.11)	32.63 (15.56)	Yes	30.20 (13.26–68.76)	<0.001
Sleep deficit	6	11.07 (4.96–24.74)	2.07 (0.70)	9.85 (4.58)	Yes	11.42 (5.10–25.59)	<0.001
Heart valve incompetence	6	17.55 (7.84–39.27)	2.27 (0.90)	15.64 (7.45)	Yes	15.98 (7.10–35.93)	<0.001
Atrial flutter	5	6.40 (2.66–15.42)	1.62 (0.10)	3.76 (0.96)	No	5.75 (2.38–13.86)	<0.001
Glucose tolerance impaired	5	6.15 (2.55–14.81)	1.60 (0.07)	3.46 (0.94)	No	6.29 (2.61–15.19)	<0.001
Craniofacial fracture	5	16.22 (6.71–39.17)	2.07 (0.54)	14.04 (6.06)	Yes	10.85 (4.47–26.31)	<0.001
Mitral valve disease	5	33.21 (13.68–80.59)	2.25 (0.73)	27.94 (12.29)	Yes	31.96 (13.06–78.20)	<0.001
Aortic valve incompetence	5	12.92 (5.36–31.19)	1.98 (0.46)	11.03 (4.55)	Yes	14.05 (5.80–34.03)	<0.001
Prostatic disorder	5	8.95 (3.71–21.56)	1.82 (0.29)	6.88 (1.14)	No	10.17 (4.20–24.59)	<0.001

Source file: table2_main_clinical_signals_revised.csv. All rows meet the primary signal criterion: a ≥ 3 reports, ROR, 95% CI, lower bound > 1, and IC_025_ > 0. PRR, and EBGM, were used as consistency measures only. Four-algorithm concordance (a ≥ 3, ROR, 95% CI, lower bound > 1, PRR ≥ 2 with χ^2^ ≥ 4, IC_025_ > 0, and EB05 > 2) is a robustness stratification and not part of the primary criterion. †aROR, exploratory adjusted reporting odds ratio from multivariable logistic regression adjusted for age category (unknown modeled as a separate category), sex, report year (centered at 2023), and healthcare-professional reporter status; model-level covariate missingness was 42.4% for age and 16.3% for sex, and HCM, severity was unavailable in FAERS., The Haldane correction (0.5 added to all cells) was applied when any cell count was zero. Four additional clinical PTs, with a = 4 (ejection fraction abnormal, sudden cardiac death, systolic dysfunction, vitamin B12 increased) are presented only in Supplementary Table S5 (sparse signals, n = 3–5); the six a = 5 PTs retained in this table also appear there for transparency. Estimates based on 3–5 reports are statistically unstable and should be interpreted with caution. PT, preferred term; ROR, reporting odds ratio; IC, information component; EBGM, empirical Bayes geometric mean; CI, confidence interval.

Cardiac-related adverse events formed the most clinically relevant signal cluster. Ejection fraction decreased was a prominent labeled cardiac safety signal (a = 99; ROR 47.08, 95% CI 38.42–57.69; IC_025_ = 4.64). Atrial fibrillation was the most frequently reported cardiac signal (a = 205; ROR 17.63, 95% CI 15.28–20.33), and cardiac failure was also detected (a = 57; ROR 5.55, 95% CI 4.27–7.22). Additional cardiac signals included left ventricular dysfunction (a = 12; ROR 14.53, 95% CI 8.22–25.69), ventricular tachycardia (a = 15; ROR 7.83, 95% CI 4.71–13.02), cardiac failure congestive (a = 12; ROR 2.65, 95% CI 1.50–4.68), and pulmonary oedema (a = 14; ROR 2.74, 95% CI 1.62–4.63). Peripheral swelling met the full three-part primary criterion (a = 57; ROR 2.21, 95% CI 1.70–2.87; IC_025_ = 0.68) and is therefore included among the primary signals. These findings are consistent with mavacamten’s pharmacological effect on cardiac contractility and its labeled risk of systolic dysfunction.

Very large disproportionality estimates were observed for LVOT-gradient- and imaging-related PTs, including transvalvular pressure gradient increased (a = 23; ROR 2742.94, 95% CI 1388.14–5420.02), acquired left ventricle outflow tract obstruction (a = 16; ROR 1546.64, 95% CI 772.73–3095.65), and echocardiogram abnormal (a = 23; ROR 144.36, 95% CI 94.02–221.66). Because obstructive HCM is defined by dynamic LVOT obstruction and mavacamten users undergo protocol-driven echocardiographic surveillance, these estimates are likely influenced by indication- and monitoring-related reporting and should not be interpreted as evidence of worsening obstruction.

Signals based on very small numbers of reports (a = 3–5) yield statistically unstable estimates despite Haldane correction and are therefore de-emphasized here; the ten sparse signals (six with a = 5 and four with a = 4) are listed in Supplementary Table S5. Consistent with this, rankings in this report emphasize signals with larger report counts, biological plausibility, and multi-algorithm consistency rather than raw ROR magnitude.

### 3.3 Administrative and product-use signals

Nine administrative/product-use and healthcare-process PTs met the primary criterion and are reported separately from clinical adverse events (Supplementary Table S4): product dose omission issue (a = 204; ROR 2.25, 95% CI 1.95–2.59), product use issue (a = 162; ROR 5.93, 95% CI 5.06–6.95), therapy interrupted (a = 52; ROR 2.01, 95% CI 1.53–2.65), treatment noncompliance (a = 28; ROR 3.23, 95% CI 2.23–4.69), product distribution issue (a = 24; ROR 8.90, 95% CI 5.95–13.32), circumstance or information capable of leading to medication error (a = 18; ROR 1.83, 95% CI 1.15–2.92), product supply issue (a = 17; ROR 8.30, 95% CI 5.14–13.38), insurance issue (a = 13; ROR 2.94, 95% CI 1.71–5.07), and product dispensing issue (a = 6; ROR 6.40, 95% CI 2.87–14.29). These terms describe healthcare-system processes rather than pharmacological toxicity and should not be interpreted as adverse physiologic effects. Three further PTs (weight increased, heart rate increased, and blood pressure increased) were classified as ambiguous because they may reflect either clinical effects or reporting/contextual artifacts (Supplementary Table S2).

### 3.4 Leading clinical signals in forest plot analysis

Figure 3 presents a forest plot of 24 clinical signals with at least five reports, prioritized by biological plausibility (cardiac, neurological, and vascular events) rather than by ROR ranking alone. The most prominent signals in this display were ejection fraction decreased, atrial fibrillation, cardiac failure, left ventricular dysfunction, and palpitations, alongside the LVOT-obstruction-related terms discussed above. Estimates for PTs with small counts (e.g., atrial flutter, a = 5) have wide confidence intervals and are shown for completeness rather than emphasis.

### 3.5 Exploratory sensitivity analysis using adjusted ROR

All 80 multivariable logistic regression models (one per primary-signal PT) converged and were classified as stable, with no separation flagged, a condition number of the covariate correlation matrix of 6.9, and a maximum variance inflation factor of 2.0 (Supplementary Table S6). After adjustment for age category, sex, report year, and healthcare-professional reporter status, 79 of the 80 primary signals retained an aROR 95% CI lower bound above 1.0 (Table 3; Supplementary Table S6). The only exception was cataract (aROR 1.54, 95% CI 0.96–2.48; p = 0.076).

**TABLE 3:** Crude versus adjusted reporting odds ratios for primary clinical signals (exploratory analysis).

Preferred term (PT)	N (a)	Crude ROR (95% CI)	aROR (95% CI)	p-value	Adjusted status
Atrial fibrillation	205	17.63 (15.28–20.33)	14.52 (12.56–16.78)	<0.001	Significant
Fatigue	184	1.62 (1.40–1.89)	1.60 (1.37–1.85)	<0.001	Significant
Dyspnoea	183	2.64 (2.27–3.07)	2.50 (2.15–2.90)	<0.001	Significant
Dizziness	169	2.83 (2.42–3.30)	2.71 (2.32–3.17)	<0.001	Significant
Ejection fraction decreased	99	47.08 (38.42–57.69)	54.74 (44.53–67.30)	<0.001	Significant
Nasopharyngitis	92	3.09 (2.51–3.81)	3.27 (2.65–4.02)	<0.001	Significant
Adverse event	85	7.51 (6.05–9.32)	7.04 (5.67–8.75)	<0.001	Significant
Palpitations	73	5.39 (4.27–6.80)	5.35 (4.24–6.76)	<0.001	Significant
Chest pain	71	3.67 (2.90–4.64)	3.69 (2.92–4.68)	<0.001	Significant
Urinary tract infection	65	2.58 (2.02–3.30)	2.25 (1.75–2.87)	<0.001	Significant
Cough	61	1.37 (1.07–1.77)	1.34 (1.04–1.73)	0.024	Significant
Peripheral swelling	57	2.21 (1.70–2.87)	2.02 (1.55–2.64)	<0.001	Significant
Cardiac failure	57	5.55 (4.27–7.22)	5.10 (3.92–6.64)	<0.001	Significant
Hypertension	56	1.95 (1.50–2.55)	1.96 (1.51–2.56)	<0.001	Significant
Hospitalisation	51	1.79 (1.36–2.36)	1.74 (1.32–2.30)	<0.001	Significant
Syncope	39	3.44 (2.51–4.72)	3.29 (2.40–4.52)	<0.001	Significant
Tooth disorder	35	13.66 (9.77–19.09)	12.59 (9.00–17.63)	<0.001	Significant
Chest discomfort	35	2.53 (1.81–3.53)	2.40 (1.71–3.37)	<0.001	Significant
Cardiac disorder	35	3.31 (2.37–4.62)	3.02 (2.16–4.22)	<0.001	Significant
Sinusitis	34	2.25 (1.60–3.15)	2.44 (1.74–3.42)	<0.001	Significant
Gastrooesophageal reflux disease	32	3.26 (2.30–4.62)	3.24 (2.29–4.60)	<0.001	Significant
Dyspepsia	30	2.40 (1.67–3.43)	2.32 (1.62–3.33)	<0.001	Significant
Arthritis	25	2.11 (1.43–3.14)	2.10 (1.41–3.11)	<0.001	Significant
Transvalvular pressure gradient increased	23	2742.94 (1388.14–5420.02)	2700.12 (1263.61–5769.68)	<0.001	Significant
Echocardiogram abnormal	23	144.36 (94.02–221.66)	181.43 (116.42–282.74)	<0.001	Significant
Rhinorrhoea	22	2.08 (1.37–3.17)	2.11 (1.38–3.21)	<0.001	Significant
Dyspnoea exertional	20	3.38 (2.18–5.25)	2.95 (1.90–4.58)	<0.001	Significant
Mitral valve incompetence	17	16.32 (10.11–26.36)	16.41 (10.13–26.57)	<0.001	Significant
Cataract	17	1.86 (1.16–3.00)	1.54 (0.96–2.48)	0.076	Non-significant
Blood pressure decreased	17	2.04 (1.26–3.28)	1.92 (1.19–3.09)	0.008	Significant
Acquired left ventricle outflow tract obstruction	16	1546.64 (772.73–3095.65)	2101.83 (973.25–4539.10)	<0.001	Significant
Ventricular tachycardia	15	7.83 (4.71–13.02)	8.03 (4.82–13.38)	<0.001	Significant
Pulmonary oedema	14	2.74 (1.62–4.63)	2.43 (1.44–4.11)	<0.001	Significant
Heart rate decreased	14	2.47 (1.46–4.18)	2.64 (1.53–4.55)	<0.001	Significant
Fluid retention	14	2.39 (1.41–4.04)	2.23 (1.32–3.77)	0.003	Significant
Fungal infection	13	2.81 (1.63–4.85)	3.02 (1.75–5.21)	<0.001	Significant
Arrhythmia	13	2.57 (1.49–4.43)	2.50 (1.45–4.31)	0.001	Significant
Cardiac failure congestive	12	2.65 (1.50–4.68)	2.31 (1.31–4.07)	0.004	Significant
Angina pectoris	12	4.59 (2.60–8.09)	4.47 (2.53–7.90)	<0.001	Significant
Left ventricular dysfunction	12	14.53 (8.22–25.69)	15.35 (8.66–27.21)	<0.001	Significant
Gout	11	4.63 (2.56–8.38)	4.43 (2.45–8.03)	<0.001	Significant
Respiratory tract congestion	11	4.94 (2.73–8.94)	4.46 (2.46–8.08)	<0.001	Significant
Blood pressure abnormal	10	3.07 (1.65–5.72)	2.76 (1.48–5.13)	0.001	Significant
Coronavirus infection	10	5.75 (3.09–10.71)	6.69 (3.59–12.48)	<0.001	Significant
Cardiovascular symptom	10	223.69 (115.13–434.61)	252.04 (126.46–502.31)	<0.001	Significant
Diverticulitis	10	2.71 (1.46–5.04)	2.38 (1.28–4.44)	0.006	Significant
Cardiomyopathy	10	6.35 (3.41–11.83)	6.47 (3.47–12.07)	<0.001	Significant
Haematuria	9	2.70 (1.40–5.19)	2.39 (1.19–4.79)	0.014	Significant
Cardiac flutter	9	11.68 (6.05–22.52)	11.08 (5.73–21.43)	<0.001	Significant
Gallbladder disorder	8	5.12 (2.56–10.27)	4.91 (2.45–9.85)	<0.001	Significant
Tooth infection	8	3.98 (1.99–7.98)	4.33 (2.16–8.68)	<0.001	Significant
Ventricular extrasystoles	8	7.54 (3.76–15.11)	7.72 (3.85–15.51)	<0.001	Significant
Stress cardiomyopathy	7	6.89 (3.28–14.49)	5.09 (2.41–10.72)	<0.001	Significant
Dizziness postural	7	4.45 (2.12–9.35)	3.93 (1.87–8.27)	<0.001	Significant
Cardiac murmur	6	9.21 (4.12–20.56)	9.35 (4.18–20.93)	<0.001	Significant
Conduction disorder	6	38.21 (16.99–85.97)	30.20 (13.26–68.76)	<0.001	Significant
Sleep deficit	6	11.07 (4.96–24.74)	11.42 (5.10–25.59)	<0.001	Significant
Heart valve incompetence	6	17.55 (7.84–39.27)	15.98 (7.10–35.93)	<0.001	Significant
Prostatic disorder	5	8.95 (3.71–21.56)	10.17 (4.20–24.59)	<0.001	Significant
Mitral valve disease	5	33.21 (13.68–80.59)	31.96 (13.06–78.20)	<0.001	Significant
Craniofacial fracture	5	16.22 (6.71–39.17)	10.85 (4.47–26.31)	<0.001	Significant
Glucose tolerance impaired	5	6.15 (2.55–14.81)	6.29 (2.61–15.19)	<0.001	Significant
Aortic valve incompetence	5	12.92 (5.36–31.19)	14.05 (5.80–34.03)	<0.001	Significant
Atrial flutter	5	6.40 (2.66–15.42)	5.75 (2.38–13.86)	<0.001	Significant
Vitamin b12 increased	4	27.88 (10.36–74.99)	25.46 (9.40–69.00)	<0.001	Significant
Systolic dysfunction	4	22.24 (8.28–59.72)	27.94 (10.34–75.50)	<0.001	Significant
Sudden cardiac death	4	22.65 (8.43–60.83)	25.31 (9.36–68.39)	<0.001	Significant
Ejection fraction abnormal	4	16.30 (6.08–43.68)	19.48 (7.23–52.46)	<0.001	Significant

Source file: table3_crude_vs_adjusted_revised.csv. aROR, adjusted reporting odds ratio from multivariable logistic regression adjusted for age category (unknown as a separate category), sex, report year (centered at 2023), and healthcare-professional reporter status; this is an exploratory adjusted analysis. All 80 models (68 clinical rows shown here; administrative/product-use and ambiguous PTs, in the supplements) converged after 13 iterations, with no separation, a condition number of the covariate correlation matrix of 6.9, and a maximum variance inflation factor of 2.0. Model-level covariate missingness was 42.4% for age and 16.3% for sex; HCM, severity is not captured in FAERS, and could not be controlled. Report counts (a) are taken from the primary signal tables; the corresponding model-based counts in Supplementary Table S6 differ by one to two reports for five PTs (fatigue, 184 versus 183; peripheral swelling, 57 versus 55; chest discomfort, 35 versus 34; heart rate decreased, 14 versus 13; haematuria, 9 versus 8), reflecting pipeline-level counting differences; the primary signal-table values are used throughout the text. Adjusted status: significant = aROR, 95% CI, lower bound > 1. Signals are listed in descending order of report count. CI, confidence interval; PT, preferred term.

**FIGURE 4:**
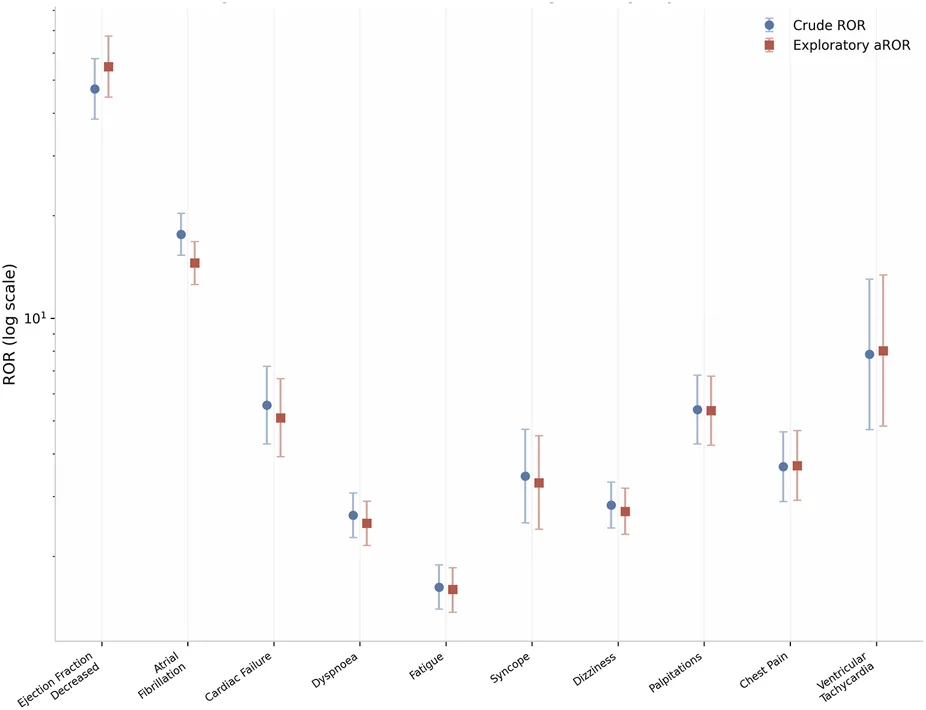
Exploratory comparison of crude and adjusted reporting odds ratios for ten selected preferred terms. Paired comparison plot showing the crude ROR and the multivariable logistic regression-adjusted ROR (aROR) for ten selected mavacamten-associated preferred terms (ejection fraction decreased, atrial fibrillation, cardiac failure, dyspnoea, fatigue, syncope, dizziness, palpitations, chest pain, and ventricular tachycardia) on a logarithmic scale. Adjusted models included age category (unknown as a separate category), sex, report year (centered at 2023), and healthcare-professional reporter status as covariates. Model-level covariate missingness was 42.4% for age and 16.3% for sex, and HCM severity was unavailable; the adjusted analysis is therefore exploratory. Crude and adjusted estimates were closely concordant for all displayed PTs, supporting the robustness of the primary disproportionality findings. ROR, reporting odds ratio; aROR, adjusted reporting odds ratio; CI, confidence interval; PT, preferred term.

The principal cardiac signals were consistent after adjustment: ejection fraction decreased (aROR 54.74, 95% CI 44.53–67.30), atrial fibrillation (aROR 14.52, 95% CI 12.56–16.78), and cardiac failure (aROR 5.10, 95% CI 3.92–6.64). Figure 4 compares crude and adjusted estimates for ten selected PTs and shows close agreement between crude and adjusted values. Given model-level missingness of 42.4% for age and 16.3% for sex, the absence of HCM severity and other clinical variables in FAERS, and the hypothesis-generating design, these adjusted estimates are exploratory and do not exclude residual confounding.

## 4 Discussion

In this FAERS pharmacovigilance analysis covering the period from 2022 Q2 to 2025 Q2 (13 quarters), we identified 2,933 unique mavacamten-associated reports among 4,514,490 deduplicated cases and detected 80 primary disproportionality signals: 68 clinical adverse events, 9 administrative/product-use and healthcare-process terms, and 3 ambiguous terms. The most clinically interpretable signals were related to the intended pharmacologic effects and known safety concerns of mavacamten, including decreased ejection fraction, left ventricular dysfunction, cardiac failure-related events, and LVOT-gradient-related preferred terms. These findings are biologically plausible given mavacamten’s mechanism as a cardiac myosin inhibitor and its known ability to reduce myocardial contractility (Green et al., 2016; Olivotto et al., 2020; U.S. Food and Drug Administration, 2022).

The prominent signal for ejection fraction decreased is particularly important. Mavacamten is designed to reduce excessive sarcomeric contractility in obstructive HCM, but excessive reduction in contractile function can lead to LVEF decline and systolic dysfunction. This risk is explicitly recognized in the FDA-approved prescribing information, which includes a boxed warning for heart failure and recommends echocardiographic monitoring of LVEF before and during therapy (U.S. Food and Drug Administration, 2022). In clinical trials, LVEF reduction was generally manageable with dose interruption or adjustment, but post-marketing data may capture broader patient populations, comorbidities, drug–drug interactions, and adherence or monitoring variability that are not fully represented in trials (Olivotto et al., 2020; Desai et al., 2022; Desai et al., 2023; U.S. Food and Drug Administration, 2022). Therefore, our findings support continued LVEF surveillance and careful dose management in real-world practice.

Signals related to LVOT gradient and transvalvular pressure gradient require nuanced interpretation. Terms such as transvalvular pressure gradient increased and acquired left ventricle outflow tract obstruction showed very large disproportionality estimates in the present analysis. These signals may reflect true residual or worsening obstruction in some patients; however, they may also be influenced by indication-related reporting, disease monitoring, or echocardiographic follow-up patterns specific to mavacamten users. Because obstructive HCM itself is defined by dynamic LVOT obstruction and because treatment decisions are guided by repeated echocardiographic measurements, reports involving gradient changes may be more likely to be submitted for mavacamten than for comparator drugs. Thus, these signals should be interpreted as reporting patterns rather than direct evidence that mavacamten causes worsening obstruction.

Atrial fibrillation also emerged as a clinically relevant cardiovascular signal. Atrial fibrillation is common in HCM and is associated with left atrial enlargement, diastolic dysfunction, heart failure symptoms, and increased thromboembolic risk (Ommen et al., 2020; Elliott et al., 2014). The observed disproportionality signal may therefore reflect a combination of background disease susceptibility, patient age and comorbidity, and potential hemodynamic changes during treatment. Although this analysis cannot determine causality, the finding is clinically meaningful because atrial fibrillation in HCM often requires active rhythm or rate control and anticoagulation assessment according to guideline-based management (Ommen et al., 2020; Elliott et al., 2014). Clinicians should remain attentive to new or worsening atrial arrhythmias in patients receiving mavacamten, particularly those with established risk factors.

Our results can be situated relative to the earlier FAERS analysis of mavacamten by Yukselen et al. (2024), which covered reports accrued from January 2022 through September 2023. To avoid mixing analysis windows, we compared estimates over the aligned post-approval period only (2022 Q2–2023 Q3; aligned rather than identical to the published calendar window): the mavacamten case counts over this aligned period were nearly identical (1,003 in the present dataset versus 1,004 unique cases in Yukselen et al.), and the key cardiac signal estimates were concordant—ejection fraction decreased, ROR 29.09 (95% CI 19.01–44.51) versus 33.60 (21.79–51.82); atrial fibrillation, 15.89 (12.32–20.48) versus 16.63 (12.72–21.75); and cardiac failure, 9.48 (6.70–13.42) versus 9.39 (6.49–13.60) (Supplementary Table S7). Differences in point estimates between the two studies are modest over the shared window and plausibly reflect differences in deduplication, drug-name mapping, and background construction. The present full-window estimates (2,933 mavacamten reports) extend rather than replace that earlier work; full-window values are reported separately and are not directly compared with the published same-window values.

The analysis also identified signals related to product use, product supply, dose omission, and insurance issues. These findings are consistent with the complexity of mavacamten prescribing and monitoring. Because mavacamten requires individualized dosing and echocardiographic surveillance, and because its use has been subject to risk mitigation measures, medication access, dispensing, monitoring logistics, and communication between patients, prescribers, pharmacies, and payers may affect real-world reporting (U.S. Food and Drug Administration, 2022). These administrative/product-use signals were therefore separated from clinical adverse events (Supplementary Table S4) and should not be interpreted as adverse physiologic effects; they nonetheless highlight implementation challenges that may influence treatment continuity and safety monitoring. Process-related PTs (therapy interrupted, product distribution issue, product dispensing issue, product supply issue, product dose omission issue, product use issue, treatment noncompliance, insurance issue, and circumstance or information capable of leading to medication error) are presented separately from clinical adverse events (Supplementary Table S4) and are not interpreted as pharmacological toxicity; this regrouping is presentational only and does not alter any PT-level estimate or the total set of 80 primary signals.

Some non-cardiovascular signals, including dental or oral-health-related preferred terms, were also observed. These signals are less readily explained by mavacamten’s known pharmacology and should be interpreted cautiously. In spontaneous reporting databases, unexpected non-cardiovascular signals may arise from reporting bias, stimulated reporting, confounding by comorbidities or concomitant medications, small cell counts, or coding variability (U.S. Food and Drug Administration, 2026; Sakaeda et al., 2013; E et al., 2001; van Puijenbroek et al., 2002; Bate et al., 1998; DuMouchel, 1999). Further evaluation using clinical records, claims data, or prospective registries would be needed before these findings could be considered clinically actionable.

Several features of the analysis support the statistical reliability of the conclusions. First, the primary signal definition combined a frequentist criterion (ROR 95% CI lower bound > 1) with a Bayesian shrinkage criterion (IC_025_ > 0), reducing the influence of small-cell random fluctuation; PRR and EBGM were computed as consistency measures, and 45 signals were concordant across all four algorithms. Second, count-threshold sensitivity analyses showed that the signal set was robust to the minimum report count (80 signals at a ≥ 3, 76 at a ≥ 5, and 58 at a ≥ 10), indicating that the main conclusions do not depend on the lowest-count stratum. Third, we deliberately de-emphasized signals based on 3–5 reports: although the Haldane correction permits estimation in the presence of zero cells, estimates in this range remain statistically unstable with wide confidence intervals, and such PTs are reported in the supplement rather than emphasized in the main text. Nonetheless, both false-positive signals (e.g., from stimulated or selective reporting) and false-negative signals (e.g., from underreporting of known labeled events) remain possible, and disproportionality results cannot exclude either.

The exploratory covariate-adjusted analysis supported the robustness of most major signals: all 80 models were stable, and 79 of 80 signals retained significance after adjustment, suggesting that the primary findings were not solely driven by measurable differences in age, sex, reporting year, or reporter characteristics. However, this analysis has important limitations. Covariate missingness was substantial at the model level (42.4% for age and 16.3% for sex); although unknown values were modeled as explicit categories rather than deleted, missingness may not be random. Only four report-level covariates were available for adjustment, and FAERS does not capture HCM severity, baseline LVEF, LVOT gradient, dose, treatment duration, comorbidities, concomitant medications, or echocardiographic monitoring frequency; adjustment therefore cannot eliminate confounding by disease severity, and residual confounding remains likely. The aROR results are accordingly presented as exploratory.

This study analyzed a single spontaneous reporting database. The status of mavacamten-associated reporting in other pharmacovigilance databases, such as EudraVigilance and VigiBase, is outside the scope of the present FAERS analysis; cross-database validation using these sources is planned as future work and no claims about their contents are made here. More broadly, linking the observed reporting signals to mechanism and clinical effect will require longitudinal data with verified exposure and outcome ascertainment, including echocardiographic parameters and HCM severity, so that reporting patterns can be distinguished from causal adverse drug reactions.

This study has several additional limitations inherent to spontaneous reporting system analyses. First, FAERS data are subject to underreporting, duplicate reporting, selective reporting, missing data, and variable report quality (U.S. Food and Drug Administration, 2026; Sakaeda et al., 2013). Second, disproportionality analyses estimate relative reporting frequency rather than incidence or absolute risk; therefore, ROR, PRR, IC, and EBGM values cannot be interpreted as measures of clinical risk (E et al., 2001; van Puijenbroek et al., 2002; Bate et al., 1998; DuMouchel, 1999). Third, causal inference is not possible because reports lack untreated comparator cohorts, validated diagnoses, standardized exposure denominators, and complete longitudinal follow-up. Fourth, confounding by indication is particularly relevant in obstructive HCM, because several reported events, such as atrial fibrillation, heart failure symptoms, LVOT obstruction, and sudden cardiac death, may also be complications of the underlying disease (Ommen et al., 2020; Elliott et al., 2014). Finally, some signals with high disproportionality estimates were based on small numbers of reports, leading to wide confidence intervals and statistical instability, and PT-level misclassification between clinical and administrative/product-use categories cannot be excluded.

Despite these limitations, this study provides a systematic post-marketing overview of mavacamten-associated adverse event reporting patterns in FAERS. The findings are broadly consistent with mavacamten’s pharmacologic profile and regulatory warnings, particularly regarding LVEF reduction and heart failure risk (Green et al., 2016; Olivotto et al., 2020; U.S. Food and Drug Administration, 2022), and with the earlier FAERS analysis by Yukselen et al. over the shared accrual window (Yukselen et al., 2024). They also identify additional cardiovascular and product-use signals that may be useful for clinicians, regulators, and researchers. Future studies using longitudinal claims data, electronic health records, registries, active surveillance systems, and other pharmacovigilance databases are needed to estimate absolute risks, account for HCM severity and treatment exposure, and clarify whether the observed signals represent causal adverse drug reactions or disease-related reporting patterns.

## 5 Conclusion

This FAERS disproportionality analysis identified 80 primary adverse event signals—68 clinical, 9 administrative/product-use, and 3 ambiguous—among 2,933 deduplicated mavacamten-associated reports from 2022 Q2 through 2025 Q2. The signal profile included expected pharmacological and cardiac safety signals, particularly decreased ejection fraction, cardiac failure, and left ventricular dysfunction, as well as clinically relevant arrhythmia signals such as atrial fibrillation. Administrative/product-use signals, reported separately, highlight the operational complexity of mavacamten treatment and monitoring. Because FAERS signals are hypothesis-generating, these findings should be confirmed in prospective registries, claims or electronic health record databases, and other pharmacovigilance systems with more complete exposure and clinical data.

## Data Availability

The original contributions presented in the study are included in the article/Supplementary Material, further inquiries can be directed to the corresponding author.

## References

[B1] Bate A. Lindquist M. Edwards I. R. Olsson S. Orre R. Lansner A. (1998). A Bayesian neural network method for adverse drug reaction signal generation. Eur. J. Clin. Pharmacol. 54 (4), 315–321. 10.1007/s002280050466 9696956

[B3] Desai M. Y. Owens A. Geske J. B. Wolski K. Naidu S. S. Smedira N. G. (2022). Myosin inhibition in patients with obstructive hypertrophic cardiomyopathy referred for septal reduction therapy. J. Am. Coll. Cardiol. 80 (2), 95–108. 10.1016/j.jacc.2022.04.048 35798455

[B4] Desai M. Y. Owens A. Wolski K. Geske J. B. Saberi S. Wang A. (2023). Mavacamten in patients with hypertrophic cardiomyopathy referred for septal reduction: week 56 results from the VALOR-HCM randomized clinical trial. JAMA Cardiol. 8 (10), 968–977. 10.1001/jamacardio.2023.3342 37639243 PMC10463171

[B5] DuMouchel W. (1999). Bayesian data mining in large frequency tables, with an application to the FDA spontaneous reporting system. Am. Statistician 53 (3), 177–190. 10.1080/00031305.1999.10474456

[B6] Evans S. J. Waller P. C. Davis S. (2001). Use of proportional reporting ratios (PRRs) for signal generation from spontaneous adverse drug reaction reports. Pharmacoepidemiol. Drug Saf. 10 (6), 483–486. 10.1002/pds.677 11828828

[B7] Elliott P. M. Anastasakis A. Borger M. A. Borggrefe M. Cecchi F. Charron P. (2014). 2014 ESC guidelines on diagnosis and management of hypertrophic cardiomyopathy. Eur. Heart J. 35 (39), 2733–2779. 10.1093/eurheartj/ehu284 25173338

[B8] Green E. M. Wakimoto H. Anderson R. L. Evanchik M. J. Gorham J. M. Harrison B. C. (2016). A small-molecule inhibitor of sarcomere contractility suppresses hypertrophic cardiomyopathy in mice. Science. 351 (6273), 617–621. 10.1126/science.aad3456 26912705 PMC4784435

[B10] Olivotto I. Oreziak A. Barriales-Villa R. Abraham T. P. Masri A. Garcia-Pavia P. (2020). Mavacamten for treatment of symptomatic obstructive hypertrophic cardiomyopathy (EXPLORER-HCM): a randomised, double-blind, placebo-controlled, phase 3 trial. Lancet 396 (10253), 759–769. 10.1016/S0140-6736(20)31792-X 32871100

[B11] Ommen S. R. Mital S. Burke M. A. Day S. M. Deswal A. Elliott P. (2020). 2020 AHA/ACC guideline for the diagnosis and treatment of patients with hypertrophic cardiomyopathy. Circulation 142 (25), e558–e631. 10.1161/CIR.0000000000000937 33215931

[B12] Sakaeda T. Tamon A. Kadoyama K. Okuno Y. (2013). Data mining of the public version of the FDA adverse event reporting system. Int. J. Med. Sci. 10 (7), 796–803. 10.7150/ijms.6048 23794943 PMC3689877

[B13] U.S. Food and Drug Administration (2022). “CAMZYOS (mavacamten) capsules, prescribing information. Initial U.S. approval: 2022,” in Updated Labeling Available Through FDA Drug Label Resources.

[B14] U.S. Food and Drug Administration (2026). FDA Adverse Event Reporting System (FAERS): Public Dashboard and Quarterly Data Files. U.S. Food and Drug Administration.

[B15] van Puijenbroek E. P. Bate A. Leufkens H. G. M. Lindquist M. Orre R. Egberts A. C. G. (2002). A comparison of measures of disproportionality for signal detection in spontaneous reporting systems for adverse drug reactions. Pharmacoepidemiol. Drug Saf. 11 (1), 3–10. 10.1002/pds.668 11998548

[B16] Yukselen A. Kocyigit D. Koseoglu T. Gurses K. M. Yenercag M. Tokgozoglu L. (2024). Post-marketing safety analysis of mavacamten using the FDA adverse event reporting system. Am. J. Cardiovasc Drugs 24 (6), 791–799. 10.1007/s40256-024-00676-0 39164512

