# Supplementary material for: Post-marketing safety signal profile of mavacamten in the FDA adverse event reporting system (FAERS): a disproportionality analysis
Source: Front Pharmacol. 2026 Sep 9;17:1906371. doi: 10.3389/fphar.2026.1906371 (PMC13597908; doi:10.3389/fphar.2026.1906371)
Supplement: Supplementary file 1 [file Table1.docx]

# Supplementary Material (revised)

Mavacamten FAERS disproportionality analysis, 2022Q2–2025Q2. All values derive from the locked v2 results. Supplementary Tables S1–S7 are presented below. There are no supplementary figures in this revision; Figures 1–4 are main-text figures.

**Table S1. All 80 primary positive signals (clinical, administrative/product-use, and ambiguous).**

| Preferred term (PT) | Class | N (a) | ROR (95% CI) | PRR (χ²) | IC (IC₀₂₅) | EBGM (EB₀₅) | Four-algorithm concordant |
| --- | --- | --- | --- | --- | --- | --- | --- |
| Atrial fibrillation | clinical | 205 | 17.63 (15.28–20.33) | 16.47 (2959.04) | 3.92 (3.72) | 16.26 (14.47) | Yes |
| Product dose omission issue | admin_product | 204 | 2.25 (1.95–2.59) | 2.16 (131.50) | 1.10 (0.89) | 2.16 (1.92) | No |
| Fatigue | clinical | 184 | 1.62 (1.40–1.89) | 1.59 (41.37) | 0.66 (0.44) | 1.47 (1.28) | No |
| Dyspnoea | clinical | 183 | 2.64 (2.27–3.07) | 2.54 (174.57) | 1.33 (1.11) | 2.53 (2.24) | Yes |
| Dizziness | clinical | 169 | 2.83 (2.42–3.30) | 2.72 (187.75) | 1.42 (1.20) | 2.71 (2.39) | Yes |
| Product use issue | admin_product | 162 | 5.93 (5.06–6.95) | 5.66 (625.55) | 2.45 (2.22) | 5.63 (4.94) | Yes |
| Ejection fraction decreased | clinical | 99 | 47.08 (38.42–57.69) | 45.53 (4190.45) | 4.94 (4.64) | 43.86 (37.06) | Yes |
| Nasopharyngitis | clinical | 92 | 3.09 (2.51–3.81) | 3.03 (125.84) | 1.56 (1.25) | 3.01 (2.52) | Yes |
| Adverse event | clinical | 85 | 7.51 (6.05–9.32) | 7.32 (463.38) | 2.76 (2.43) | 7.25 (6.04) | Yes |
| Palpitations | clinical | 73 | 5.39 (4.27–6.80) | 5.28 (253.68) | 2.31 (1.96) | 5.23 (4.30) | Yes |
| Chest pain | clinical | 71 | 3.67 (2.90–4.64) | 3.60 (134.03) | 1.79 (1.43) | 3.57 (2.93) | Yes |
| Weight increased | ambiguous | 69 | 2.36 (1.86–3.00) | 2.33 (52.79) | 1.18 (0.82) | 2.31 (1.88) | No |
| Urinary tract infection | clinical | 65 | 2.58 (2.02–3.30) | 2.54 (61.31) | 1.30 (0.93) | 2.52 (2.05) | Yes |
| Cough | clinical | 61 | 1.37 (1.07–1.77) | 1.37 (6.07) | 0.43 (0.04) | 1.17 (1.00) | No |
| Peripheral swelling | clinical | 57 | 2.21 (1.70–2.87) | 2.18 (36.79) | 1.08 (0.68) | 2.13 (1.62) | No |
| Cardiac failure | clinical | 57 | 5.55 (4.27–7.22) | 5.46 (207.89) | 2.33 (1.93) | 5.40 (4.32) | Yes |
| Hypertension | clinical | 56 | 1.95 (1.50–2.55) | 1.94 (25.54) | 0.92 (0.51) | 1.74 (1.22) | No |
| Therapy interrupted | admin_product | 52 | 2.01 (1.53–2.65) | 1.99 (25.96) | 0.95 (0.54) | 1.81 (1.24) | No |
| Hospitalisation | clinical | 51 | 1.79 (1.36–2.36) | 1.77 (17.40) | 0.79 (0.37) | 1.43 (1.10) | No |
| Syncope | clinical | 39 | 3.44 (2.51–4.72) | 3.41 (66.49) | 1.66 (1.18) | 3.36 (2.56) | Yes |
| Blood pressure increased | ambiguous | 37 | 1.71 (1.23–2.36) | 1.70 (10.70) | 0.72 (0.22) | 1.27 (1.01) | No |
| Tooth disorder | clinical | 35 | 13.66 (9.77–19.09) | 13.51 (402.27) | 3.30 (2.78) | 13.19 (9.89) | Yes |
| Cardiac disorder | clinical | 35 | 3.31 (2.37–4.62) | 3.28 (55.65) | 1.60 (1.09) | 3.23 (2.43) | Yes |
| Chest discomfort | clinical | 35 | 2.53 (1.81–3.53) | 2.51 (31.98) | 1.25 (0.73) | 2.43 (1.72) | No |
| Sinusitis | clinical | 34 | 2.25 (1.60–3.15) | 2.23 (23.20) | 1.09 (0.56) | 1.99 (1.20) | No |
| Gastrooesophageal reflux disease | clinical | 32 | 3.26 (2.30–4.62) | 3.24 (49.50) | 1.58 (1.03) | 3.18 (2.35) | Yes |
| Dyspepsia | clinical | 30 | 2.40 (1.67–3.43) | 2.38 (24.12) | 1.16 (0.60) | 2.16 (1.22) | No |
| Treatment noncompliance | admin_product | 28 | 3.23 (2.23–4.69) | 3.21 (42.60) | 1.55 (0.97) | 3.14 (2.27) | Yes |
| Heart rate increased | ambiguous | 25 | 1.96 (1.32–2.91) | 1.95 (11.68) | 0.89 (0.26) | 1.34 (0.99) | No |
| Arthritis | clinical | 25 | 2.11 (1.43–3.14) | 2.11 (14.55) | 0.98 (0.36) | 1.51 (1.03) | No |
| Product distribution issue | admin_product | 24 | 8.90 (5.95–13.32) | 8.84 (166.08) | 2.72 (2.08) | 8.61 (6.07) | Yes |
| Echocardiogram abnormal | clinical | 23 | 144.36 (94.02–221.66) | 143.23 (2971.99) | 4.32 (3.67) | 121.77 (85.12) | Yes |
| Transvalvular pressure gradient increased | clinical | 23 | 2742.94 (1388.14–5420.02) | 2721.44 (22586.65) | 4.52 (3.87) | 676.21 (472.69) | Yes |
| Rhinorrhoea | clinical | 22 | 2.08 (1.37–3.17) | 2.07 (12.27) | 0.95 (0.29) | 1.39 (0.99) | No |
| Dyspnoea exertional | clinical | 20 | 3.38 (2.18–5.25) | 3.36 (33.18) | 1.56 (0.86) | 3.23 (2.14) | Yes |
| Circumstance or information capable of leading to medication error | admin_product | 18 | 1.83 (1.15–2.92) | 1.83 (6.79) | 0.77 (0.03) | 1.18 (0.92) | No |
| Blood pressure decreased | clinical | 17 | 2.04 (1.26–3.28) | 2.03 (8.92) | 0.90 (0.13) | 1.25 (0.94) | No |
| Cataract | clinical | 17 | 1.86 (1.16–3.00) | 1.86 (6.75) | 0.79 (0.02) | 1.18 (0.92) | No |
| Product supply issue | admin_product | 17 | 8.30 (5.14–13.38) | 8.25 (107.88) | 2.51 (1.74) | 7.97 (5.24) | Yes |
| Mitral valve incompetence | clinical | 17 | 16.32 (10.11–26.36) | 16.23 (240.54) | 3.09 (2.32) | 15.53 (10.20) | Yes |
| Acquired left ventricle outflow tract obstruction | clinical | 16 | 1546.64 (772.73–3095.65) | 1538.21 (12289.74) | 4.01 (3.22) | 505.96 (327.90) | Yes |
| Ventricular tachycardia | clinical | 15 | 7.83 (4.71–13.02) | 7.79 (88.46) | 2.40 (1.58) | 7.50 (4.79) | Yes |
| Fluid retention | clinical | 14 | 2.39 (1.41–4.04) | 2.38 (11.26) | 1.08 (0.22) | 1.41 (0.94) | No |
| Pulmonary oedema | clinical | 14 | 2.74 (1.62–4.63) | 2.73 (15.32) | 1.24 (0.39) | 1.83 (0.99) | No |
| Heart rate decreased | clinical | 14 | 2.47 (1.46–4.18) | 2.47 (12.21) | 1.12 (0.26) | 1.49 (0.95) | No |
| Arrhythmia | clinical | 13 | 2.57 (1.49–4.43) | 2.56 (12.39) | 1.15 (0.26) | 1.53 (0.95) | No |
| Insurance issue | admin_product | 13 | 2.94 (1.71–5.07) | 2.93 (16.55) | 1.31 (0.42) | 2.03 (1.00) | No |
| Fungal infection | clinical | 13 | 2.81 (1.63–4.85) | 2.80 (15.09) | 1.26 (0.37) | 1.83 (0.98) | No |
| Cardiac failure congestive | clinical | 12 | 2.65 (1.50–4.68) | 2.65 (12.30) | 1.17 (0.24) | 1.54 (0.94) | No |
| Left ventricular dysfunction | clinical | 12 | 14.53 (8.22–25.69) | 14.48 (149.19) | 2.77 (1.84) | 13.68 (8.25) | Yes |
| Angina pectoris | clinical | 12 | 4.59 (2.60–8.09) | 4.57 (33.42) | 1.78 (0.85) | 4.27 (2.44) | Yes |
| Respiratory tract congestion | clinical | 11 | 4.94 (2.73–8.94) | 4.93 (34.33) | 1.83 (0.85) | 4.58 (2.55) | Yes |
| Gout | clinical | 11 | 4.63 (2.56–8.38) | 4.62 (31.12) | 1.76 (0.79) | 4.22 (2.18) | Yes |
| Diverticulitis | clinical | 10 | 2.71 (1.46–5.04) | 2.70 (10.73) | 1.16 (0.13) | 1.43 (0.91) | No |
| Coronavirus infection | clinical | 10 | 5.75 (3.09–10.71) | 5.73 (38.95) | 1.93 (0.90) | 5.35 (2.98) | Yes |
| Cardiomyopathy | clinical | 10 | 6.35 (3.41–11.83) | 6.33 (44.72) | 2.02 (0.99) | 5.95 (3.37) | Yes |
| Cardiovascular symptom | clinical | 10 | 223.69 (115.13–434.61) | 222.93 (1929.70) | 3.32 (2.29) | 156.22 (89.38) | Yes |
| Blood pressure abnormal | clinical | 10 | 3.07 (1.65–5.72) | 3.06 (13.88) | 1.30 (0.27) | 1.77 (0.94) | No |
| Cardiac flutter | clinical | 9 | 11.68 (6.05–22.52) | 11.64 (86.93) | 2.42 (1.32) | 10.88 (6.02) | Yes |
| Haematuria | clinical | 9 | 2.70 (1.40–5.19) | 2.69 (9.57) | 1.13 (0.04) | 1.35 (0.90) | No |
| Gallbladder disorder | clinical | 8 | 5.12 (2.56–10.27) | 5.11 (26.39) | 1.73 (0.56) | 4.15 (1.12) | No |
| Tooth infection | clinical | 8 | 3.98 (1.99–7.98) | 3.98 (17.79) | 1.49 (0.33) | 2.49 (0.96) | No |
| Ventricular extrasystoles | clinical | 8 | 7.54 (3.76–15.11) | 7.52 (45.00) | 2.04 (0.87) | 6.91 (3.60) | Yes |
| Stress cardiomyopathy | clinical | 7 | 6.89 (3.28–14.49) | 6.88 (35.00) | 1.89 (0.63) | 5.96 (2.36) | Yes |
| Dizziness postural | clinical | 7 | 4.45 (2.12–9.35) | 4.44 (18.61) | 1.54 (0.28) | 2.71 (0.96) | No |
| Sleep deficit | clinical | 6 | 11.07 (4.96–24.74) | 11.05 (54.48) | 2.07 (0.70) | 9.85 (4.58) | Yes |
| Cardiac murmur | clinical | 6 | 9.21 (4.12–20.56) | 9.19 (43.56) | 1.97 (0.60) | 8.01 (3.48) | Yes |
| Product dispensing issue | admin_product | 6 | 6.40 (2.87–14.29) | 6.39 (27.18) | 1.74 (0.37) | 4.74 (1.05) | No |
| Heart valve incompetence | clinical | 6 | 17.55 (7.84–39.27) | 17.51 (92.38) | 2.27 (0.90) | 15.64 (7.45) | Yes |
| Conduction disorder | clinical | 6 | 38.21 (16.99–85.97) | 38.14 (211.74) | 2.48 (1.11) | 32.63 (15.56) | Yes |
| Mitral valve disease | clinical | 5 | 33.21 (13.68–80.59) | 33.15 (152.62) | 2.25 (0.73) | 27.94 (12.29) | Yes |
| Craniofacial fracture | clinical | 5 | 16.22 (6.71–39.17) | 16.19 (70.53) | 2.07 (0.54) | 14.04 (6.06) | Yes |
| Prostatic disorder | clinical | 5 | 8.95 (3.71–21.56) | 8.93 (35.03) | 1.82 (0.29) | 6.88 (1.14) | No |
| Aortic valve incompetence | clinical | 5 | 12.92 (5.36–31.19) | 12.90 (54.46) | 1.98 (0.46) | 11.03 (4.55) | Yes |
| Atrial flutter | clinical | 5 | 6.40 (2.66–15.42) | 6.39 (22.66) | 1.62 (0.10) | 3.76 (0.96) | No |
| Glucose tolerance impaired | clinical | 5 | 6.15 (2.55–14.81) | 6.14 (21.43) | 1.60 (0.07) | 3.46 (0.94) | No |
| Ejection fraction abnormal | clinical | 4 | 16.30 (6.08–43.68) | 16.28 (56.76) | 1.85 (0.11) | 12.90 (4.18) | Yes |
| Sudden cardiac death | clinical | 4 | 22.65 (8.43–60.83) | 22.62 (81.47) | 1.93 (0.20) | 18.47 (7.00) | Yes |
| Systolic dysfunction | clinical | 4 | 22.24 (8.28–59.72) | 22.21 (79.88) | 1.93 (0.19) | 18.13 (6.85) | Yes |
| Vitamin b12 increased | clinical | 4 | 27.88 (10.36–74.99) | 27.84 (101.67) | 1.97 (0.24) | 22.67 (8.78) | Yes |

*Note.* All rows meet the primary signal criterion: a ≥ 3, ROR 95% CI lower bound > 1, and IC₀₂₅ > 0. PRR and EBGM are consistency measures only; four-algorithm concordance (a ≥ 3, ROR 95% CI lower bound > 1, PRR ≥ 2 with χ² ≥ 4, IC₀₂₅ > 0, EB05 > 2; n = 45) is a robustness stratification and not part of the primary criterion. Haldane correction (0.5 added to all cells) was applied when any cell count was zero. Class: clinical = clinical adverse event; admin_product = administrative/product-use term; ambiguous = ambiguous term. PT = preferred term; ROR = reporting odds ratio; PRR = proportional reporting ratio; IC = information component; EBGM = empirical Bayes geometric mean; CI = confidence interval.

**Table S2. Ambiguous preferred terms among the primary signals (n = 3).**

| Preferred term (PT) | N (a) | ROR (95% CI) | IC (IC₀₂₅) | aROR (95% CI) | p-value |
| --- | --- | --- | --- | --- | --- |
| Weight increased | 69 | 2.36 (1.86–3.00) | 1.18 (0.82) | 2.91 (2.29–3.70) | <0.001 |
| Heart rate increased | 25 | 1.96 (1.32–2.91) | 0.89 (0.26) | 2.06 (1.39–3.05) | <0.001 |
| Blood pressure increased | 37 | 1.71 (1.23–2.36) | 0.72 (0.22) | 1.64 (1.19–2.27) | 0.003 |

*Note.* aROR = exploratory adjusted reporting odds ratio (see Table 3 footnote for covariates, missingness, and limitations). PT = preferred term; ROR = reporting odds ratio; IC = information component; CI = confidence interval.

**Table S3. Count-threshold sensitivity analysis of the primary signal criterion.**

| Count threshold | Total signals | Clinical | Administrative/product-use | Ambiguous |
| --- | --- | --- | --- | --- |
| a>=3 | 80 | 68 | 9 | 3 |
| a>=5 | 76 | 64 | 9 | 3 |
| a>=10 | 58 | 47 | 8 | 3 |

*Note.* Primary criterion (a ≥ 3, ROR 95% CI lower bound > 1, IC₀₂₅ > 0) re-applied at minimum report counts of a ≥ 5 and a ≥ 10.

**Table S4. Administrative/product-use and healthcare-process signals (n = 9; reported separately from clinical adverse events).**

| Preferred term (PT) | N (a) | ROR (95% CI) | IC (IC₀₂₅) |
| --- | --- | --- | --- |
| Product dose omission issue | 204 | 2.25 (1.95–2.59) | 1.10 (0.89) |
| Product use issue | 162 | 5.93 (5.06–6.95) | 2.45 (2.22) |
| Therapy interrupted | 52 | 2.01 (1.53–2.65) | 0.95 (0.54) |
| Treatment noncompliance | 28 | 3.23 (2.23–4.69) | 1.55 (0.97) |
| Product distribution issue | 24 | 8.90 (5.95–13.32) | 2.72 (2.08) |
| Circumstance or information capable of leading to medication error | 18 | 1.83 (1.15–2.92) | 0.77 (0.03) |
| Product supply issue | 17 | 8.30 (5.14–13.38) | 2.51 (1.74) |
| Insurance issue | 13 | 2.94 (1.71–5.07) | 1.31 (0.42) |
| Product dispensing issue | 6 | 6.40 (2.87–14.29) | 1.74 (0.37) |

*Note.* These PTs describe healthcare-system processes rather than pharmacological toxicity. All rows meet the primary signal criterion (a ≥ 3, ROR 95% CI lower bound > 1, IC₀₂₅ > 0). PT = preferred term; ROR = reporting odds ratio; IC = information component; CI = confidence interval.

**Table S5. Sparse signals based on 3–5 reports (n = 10).**

| Preferred term (PT) | N (a) | ROR (95% CI) | IC (IC₀₂₅) | Four-algorithm concordant | aROR (95% CI) | p-value |
| --- | --- | --- | --- | --- | --- | --- |
| Mitral valve disease | 5 | 33.21 (13.68–80.59) | 2.25 (0.73) | Yes | 31.96 (13.06–78.20) | <0.001 |
| Craniofacial fracture | 5 | 16.22 (6.71–39.17) | 2.07 (0.54) | Yes | 10.85 (4.47–26.31) | <0.001 |
| Glucose tolerance impaired | 5 | 6.15 (2.55–14.81) | 1.60 (0.07) | No | 6.29 (2.61–15.19) | <0.001 |
| Atrial flutter | 5 | 6.40 (2.66–15.42) | 1.62 (0.10) | No | 5.75 (2.38–13.86) | <0.001 |
| Prostatic disorder | 5 | 8.95 (3.71–21.56) | 1.82 (0.29) | No | 10.17 (4.20–24.59) | <0.001 |
| Aortic valve incompetence | 5 | 12.92 (5.36–31.19) | 1.98 (0.46) | Yes | 14.05 (5.80–34.03) | <0.001 |
| Vitamin b12 increased | 4 | 27.88 (10.36–74.99) | 1.97 (0.24) | Yes | 25.46 (9.40–69.00) | <0.001 |
| Ejection fraction abnormal | 4 | 16.30 (6.08–43.68) | 1.85 (0.11) | Yes | 19.48 (7.23–52.46) | <0.001 |
| Sudden cardiac death | 4 | 22.65 (8.43–60.83) | 1.93 (0.20) | Yes | 25.31 (9.36–68.39) | <0.001 |
| Systolic dysfunction | 4 | 22.24 (8.28–59.72) | 1.93 (0.19) | Yes | 27.94 (10.34–75.50) | <0.001 |

*Note.* Estimates based on 3–5 reports are statistically unstable despite Haldane correction and are presented in the supplement only, without emphasis in the main text. All rows meet the primary signal criterion. aROR = exploratory adjusted reporting odds ratio. PT = preferred term; ROR = reporting odds ratio; IC = information component; CI = confidence interval.

**Table S6. Model diagnostics for the 80 exploratory adjusted-ROR models.**

| PT | Mavacamten N with PT | Age missing (%) | Sex missing (%) | Converged | Iterations | Separation | Max VIF | Condition number | aROR (95% CI) | p-value | Model status |
| --- | --- | --- | --- | --- | --- | --- | --- | --- | --- | --- | --- |
| Fatigue | 183 | 42.4 | 16.3 | Yes | 13 | No | 2.0 | 6.9 | 1.60 (1.37–1.85) | <0.001 | stable |
| Product dose omission issue | 204 | 42.4 | 16.3 | Yes | 13 | No | 2.0 | 6.9 | 2.44 (2.11–2.82) | <0.001 | stable |
| Dyspnoea | 183 | 42.4 | 16.3 | Yes | 13 | No | 2.0 | 6.9 | 2.50 (2.15–2.90) | <0.001 | stable |
| Dizziness | 169 | 42.4 | 16.3 | Yes | 13 | No | 2.0 | 6.9 | 2.71 (2.32–3.17) | <0.001 | stable |
| Cough | 61 | 42.4 | 16.3 | Yes | 13 | No | 2.0 | 6.9 | 1.34 (1.04–1.73) | 0.024 | stable |
| Nasopharyngitis | 92 | 42.4 | 16.3 | Yes | 13 | No | 2.0 | 6.9 | 3.27 (2.65–4.02) | <0.001 | stable |
| Weight increased | 69 | 42.4 | 16.3 | Yes | 13 | No | 2.0 | 6.9 | 2.91 (2.29–3.70) | <0.001 | stable |
| Hypertension | 56 | 42.4 | 16.3 | Yes | 13 | No | 2.0 | 6.9 | 1.96 (1.51–2.56) | <0.001 | stable |
| Hospitalisation | 51 | 42.4 | 16.3 | Yes | 13 | No | 2.0 | 6.9 | 1.74 (1.32–2.30) | <0.001 | stable |
| Product use issue | 162 | 42.4 | 16.3 | Yes | 13 | No | 2.0 | 6.9 | 6.59 (5.62–7.73) | <0.001 | stable |
| Therapy interrupted | 52 | 42.4 | 16.3 | Yes | 13 | No | 2.0 | 6.9 | 1.73 (1.31–2.27) | <0.001 | stable |
| Peripheral swelling | 55 | 42.4 | 16.3 | Yes | 13 | No | 2.0 | 6.9 | 2.02 (1.55–2.64) | <0.001 | stable |
| Urinary tract infection | 65 | 42.4 | 16.3 | Yes | 13 | No | 2.0 | 6.9 | 2.25 (1.75–2.87) | <0.001 | stable |
| Blood pressure increased | 37 | 42.4 | 16.3 | Yes | 13 | No | 2.0 | 6.9 | 1.64 (1.19–2.27) | 0.003 | stable |
| Chest pain | 71 | 42.4 | 16.3 | Yes | 13 | No | 2.0 | 6.9 | 3.69 (2.92–4.68) | <0.001 | stable |
| Sinusitis | 34 | 42.4 | 16.3 | Yes | 13 | No | 2.0 | 6.9 | 2.44 (1.74–3.42) | <0.001 | stable |
| Chest discomfort | 34 | 42.4 | 16.3 | Yes | 13 | No | 2.0 | 6.9 | 2.40 (1.71–3.37) | <0.001 | stable |
| Palpitations | 73 | 42.4 | 16.3 | Yes | 13 | No | 2.0 | 6.9 | 5.35 (4.24–6.76) | <0.001 | stable |
| Heart rate increased | 25 | 42.4 | 16.3 | Yes | 13 | No | 2.0 | 6.9 | 2.06 (1.39–3.05) | <0.001 | stable |
| Atrial fibrillation | 205 | 42.4 | 16.3 | Yes | 13 | No | 2.0 | 6.9 | 14.52 (12.56–16.78) | <0.001 | stable |
| Dyspepsia | 30 | 42.4 | 16.3 | Yes | 13 | No | 2.0 | 6.9 | 2.32 (1.62–3.33) | <0.001 | stable |
| Arthritis | 25 | 42.4 | 16.3 | Yes | 13 | No | 2.0 | 6.9 | 2.10 (1.41–3.11) | <0.001 | stable |
| Adverse event | 85 | 42.4 | 16.3 | Yes | 13 | No | 2.0 | 6.9 | 7.04 (5.67–8.75) | <0.001 | stable |
| Syncope | 39 | 42.4 | 16.3 | Yes | 13 | No | 2.0 | 6.9 | 3.29 (2.40–4.52) | <0.001 | stable |
| Cardiac disorder | 35 | 42.4 | 16.3 | Yes | 13 | No | 2.0 | 6.9 | 3.02 (2.16–4.22) | <0.001 | stable |
| Rhinorrhoea | 22 | 42.4 | 16.3 | Yes | 13 | No | 2.0 | 6.9 | 2.11 (1.38–3.21) | <0.001 | stable |
| Cardiac failure | 57 | 42.4 | 16.3 | Yes | 13 | No | 2.0 | 6.9 | 5.10 (3.92–6.64) | <0.001 | stable |
| Circumstance or information capable of leading to medication error | 18 | 42.4 | 16.3 | Yes | 13 | No | 2.0 | 6.9 | 2.25 (1.41–3.58) | <0.001 | stable |
| Gastrooesophageal reflux disease | 32 | 42.4 | 16.3 | Yes | 13 | No | 2.0 | 6.9 | 3.24 (2.29–4.60) | <0.001 | stable |
| Cataract | 17 | 42.4 | 16.3 | Yes | 13 | No | 2.0 | 6.9 | 1.54 (0.96–2.48) | 0.076 | stable |
| Treatment noncompliance | 28 | 42.4 | 16.3 | Yes | 13 | No | 2.0 | 6.9 | 3.42 (2.36–4.97) | <0.001 | stable |
| Blood pressure decreased | 17 | 42.4 | 16.3 | Yes | 13 | No | 2.0 | 6.9 | 1.92 (1.19–3.09) | 0.008 | stable |
| Dyspnoea exertional | 20 | 42.4 | 16.3 | Yes | 13 | No | 2.0 | 6.9 | 2.95 (1.90–4.58) | <0.001 | stable |
| Fluid retention | 14 | 42.4 | 16.3 | Yes | 13 | No | 2.0 | 6.9 | 2.23 (1.32–3.77) | 0.003 | stable |
| Heart rate decreased | 13 | 42.4 | 16.3 | Yes | 13 | No | 2.0 | 6.9 | 2.64 (1.53–4.55) | <0.001 | stable |
| Pulmonary oedema | 14 | 42.4 | 16.3 | Yes | 13 | No | 2.0 | 6.9 | 2.43 (1.44–4.11) | <0.001 | stable |
| Arrhythmia | 13 | 42.4 | 16.3 | Yes | 13 | No | 2.0 | 6.9 | 2.50 (1.45–4.31) | 0.001 | stable |
| Fungal infection | 13 | 42.4 | 16.3 | Yes | 13 | No | 2.0 | 6.9 | 3.02 (1.75–5.21) | <0.001 | stable |
| Cardiac failure congestive | 12 | 42.4 | 16.3 | Yes | 13 | No | 2.0 | 6.9 | 2.31 (1.31–4.07) | 0.004 | stable |
| Insurance issue | 13 | 42.4 | 16.3 | Yes | 13 | No | 2.0 | 6.9 | 3.04 (1.76–5.24) | <0.001 | stable |
| Diverticulitis | 10 | 42.4 | 16.3 | Yes | 13 | No | 2.0 | 6.9 | 2.38 (1.28–4.44) | 0.006 | stable |
| Haematuria | 8 | 42.4 | 16.3 | Yes | 13 | No | 2.0 | 6.9 | 2.39 (1.19–4.79) | 0.014 | stable |
| Blood pressure abnormal | 10 | 42.4 | 16.3 | Yes | 13 | No | 2.0 | 6.9 | 2.76 (1.48–5.13) | 0.001 | stable |
| Product distribution issue | 24 | 42.4 | 16.3 | Yes | 13 | No | 2.0 | 6.9 | 7.17 (4.79–10.75) | <0.001 | stable |
| Angina pectoris | 12 | 42.4 | 16.3 | Yes | 13 | No | 2.0 | 6.9 | 4.47 (2.53–7.90) | <0.001 | stable |
| Tooth disorder | 35 | 42.4 | 16.3 | Yes | 13 | No | 2.0 | 6.9 | 12.59 (9.00–17.63) | <0.001 | stable |
| Gout | 11 | 42.4 | 16.3 | Yes | 13 | No | 2.0 | 6.9 | 4.43 (2.45–8.03) | <0.001 | stable |
| Respiratory tract congestion | 11 | 42.4 | 16.3 | Yes | 13 | No | 2.0 | 6.9 | 4.46 (2.46–8.08) | <0.001 | stable |
| Ejection fraction decreased | 99 | 42.4 | 16.3 | Yes | 13 | No | 2.0 | 6.9 | 54.74 (44.53–67.30) | <0.001 | stable |
| Product supply issue | 17 | 42.4 | 16.3 | Yes | 13 | No | 2.0 | 6.9 | 7.92 (4.90–12.80) | <0.001 | stable |
| Tooth infection | 8 | 42.4 | 16.3 | Yes | 13 | No | 2.0 | 6.9 | 4.33 (2.16–8.68) | <0.001 | stable |
| Ventricular tachycardia | 15 | 42.4 | 16.3 | Yes | 13 | No | 2.0 | 6.9 | 8.03 (4.82–13.38) | <0.001 | stable |
| Coronavirus infection | 10 | 42.4 | 16.3 | Yes | 13 | No | 2.0 | 6.9 | 6.69 (3.59–12.48) | <0.001 | stable |
| Cardiomyopathy | 10 | 42.4 | 16.3 | Yes | 13 | No | 2.0 | 6.9 | 6.47 (3.47–12.07) | <0.001 | stable |
| Dizziness postural | 7 | 42.4 | 16.3 | Yes | 13 | No | 2.0 | 6.9 | 3.93 (1.87–8.27) | <0.001 | stable |
| Gallbladder disorder | 8 | 42.4 | 16.3 | Yes | 13 | No | 2.0 | 6.9 | 4.91 (2.45–9.85) | <0.001 | stable |
| Ventricular extrasystoles | 8 | 42.4 | 16.3 | Yes | 13 | No | 2.0 | 6.9 | 7.72 (3.85–15.51) | <0.001 | stable |
| Mitral valve incompetence | 17 | 42.4 | 16.3 | Yes | 13 | No | 2.0 | 6.9 | 16.41 (10.13–26.57) | <0.001 | stable |
| Stress cardiomyopathy | 7 | 42.4 | 16.3 | Yes | 13 | No | 2.0 | 6.9 | 5.09 (2.41–10.72) | <0.001 | stable |
| Product dispensing issue | 6 | 42.4 | 16.3 | Yes | 13 | No | 2.0 | 6.9 | 4.85 (2.17–10.83) | <0.001 | stable |
| Left ventricular dysfunction | 12 | 42.4 | 16.3 | Yes | 13 | No | 2.0 | 6.9 | 15.35 (8.66–27.21) | <0.001 | stable |
| Glucose tolerance impaired | 5 | 42.4 | 16.3 | Yes | 13 | No | 2.0 | 6.9 | 6.29 (2.61–15.19) | <0.001 | stable |
| Atrial flutter | 5 | 42.4 | 16.3 | Yes | 13 | No | 2.0 | 6.9 | 5.75 (2.38–13.86) | <0.001 | stable |
| Cardiac flutter | 9 | 42.4 | 16.3 | Yes | 13 | No | 2.0 | 6.9 | 11.08 (5.73–21.43) | <0.001 | stable |
| Cardiac murmur | 6 | 42.4 | 16.3 | Yes | 13 | No | 2.0 | 6.9 | 9.35 (4.18–20.93) | <0.001 | stable |
| Prostatic disorder | 5 | 42.4 | 16.3 | Yes | 13 | No | 2.0 | 6.9 | 10.17 (4.20–24.59) | <0.001 | stable |
| Sleep deficit | 6 | 42.4 | 16.3 | Yes | 13 | No | 2.0 | 6.9 | 11.42 (5.10–25.59) | <0.001 | stable |
| Aortic valve incompetence | 5 | 42.4 | 16.3 | Yes | 13 | No | 2.0 | 6.9 | 14.05 (5.80–34.03) | <0.001 | stable |
| Heart valve incompetence | 6 | 42.4 | 16.3 | Yes | 13 | No | 2.0 | 6.9 | 15.98 (7.10–35.93) | <0.001 | stable |
| Craniofacial fracture | 5 | 42.4 | 16.3 | Yes | 13 | No | 2.0 | 6.9 | 10.85 (4.47–26.31) | <0.001 | stable |
| Ejection fraction abnormal | 4 | 42.4 | 16.3 | Yes | 13 | No | 2.0 | 6.9 | 19.48 (7.23–52.46) | <0.001 | stable |
| Systolic dysfunction | 4 | 42.4 | 16.3 | Yes | 13 | No | 2.0 | 6.9 | 27.94 (10.34–75.50) | <0.001 | stable |
| Sudden cardiac death | 4 | 42.4 | 16.3 | Yes | 13 | No | 2.0 | 6.9 | 25.31 (9.36–68.39) | <0.001 | stable |
| Echocardiogram abnormal | 23 | 42.4 | 16.3 | Yes | 13 | No | 2.0 | 6.9 | 181.43 (116.42–282.74) | <0.001 | stable |
| Conduction disorder | 6 | 42.4 | 16.3 | Yes | 13 | No | 2.0 | 6.9 | 30.20 (13.26–68.76) | <0.001 | stable |
| Mitral valve disease | 5 | 42.4 | 16.3 | Yes | 13 | No | 2.0 | 6.9 | 31.96 (13.06–78.20) | <0.001 | stable |
| Vitamin b12 increased | 4 | 42.4 | 16.3 | Yes | 13 | No | 2.0 | 6.9 | 25.46 (9.40–69.00) | <0.001 | stable |
| Cardiovascular symptom | 10 | 42.4 | 16.3 | Yes | 13 | No | 2.0 | 6.9 | 252.04 (126.46–502.31) | <0.001 | stable |
| Transvalvular pressure gradient increased | 23 | 42.4 | 16.3 | Yes | 13 | No | 2.0 | 6.9 | 2700.12 (1263.61–5769.68) | <0.001 | stable |
| Acquired left ventricle outflow tract obstruction | 16 | 42.4 | 16.3 | Yes | 13 | No | 2.0 | 6.9 | 2101.83 (973.25–4539.10) | <0.001 | stable |

*Note.* One logistic regression model was fitted per primary-signal PT; model N = 4,514,490 and mavacamten N = 2,933 for all models. Covariates: age category (unknown as separate category), sex, report year (centered at 2023), healthcare-professional reporter status. The aROR analysis is exploratory; model-level missingness was 42.4% for age and 16.3% for sex, and HCM severity was unavailable in FAERS. All 80 models converged (13 iterations), with no separation flagged, condition number of the covariate correlation matrix 6.9, and maximum variance inflation factor 2.0; 79 of 80 models yielded a significant aROR (exception: cataract, p = 0.076). Model-based mavacamten PT counts differ from primary signal-table counts by one to two reports for five PTs (fatigue, 183 versus 184; peripheral swelling, 55 versus 57; chest discomfort, 34 versus 35; heart rate decreased, 13 versus 14; haematuria, 8 versus 9); the primary signal-table values are used in the text. PT = preferred term; VIF = variance inflation factor; aROR = adjusted reporting odds ratio; CI = confidence interval.

**Table S7. Comparison with Yukselen et al. 2024 over the aligned post-approval period (2022 Q2–2023 Q3).**

| PT | Yukselen 2024 ROR (95% CI) | Yukselen IC (IC₀₂₅) | Same-window a | Same-window ROR (95% CI) | Same-window IC (IC₀₂₅) | Full-window a | Full-window ROR (95% CI) | Full-window IC (IC₀₂₅) | aROR (95% CI) |
| --- | --- | --- | --- | --- | --- | --- | --- | --- | --- |
| Ejection fraction decreased | 33.6 (21.79–51.82) | 5.03 (4.61) | 22 | 29.09 (19.01–44.51) | 3.66 (2.99) | 99 | 47.08 (38.42–57.69) | 4.94 (4.64) | 54.74 (44.53–67.3) |
| Atrial fibrillation | 16.63 (12.72–21.75) | 3.97 (3.71) | 64 | 15.89 (12.32–20.48) | 3.6 (3.23) | 205 | 17.63 (15.28–20.33) | 3.92 (3.72) | 14.52 (12.56–16.78) |
| Cardiac failure | 9.39 (6.49–13.60) | 3.19 (2.83) | 33 | 9.48 (6.70–13.42) | 2.86 (2.33) | 57 | 5.55 (4.27–7.22) | 2.33 (1.93) | 5.1 (3.92–6.64) |

*Note.* Yukselen columns are the published values from Yukselen et al. (Am J Cardiovasc Drugs. 2024;24(6):791–799; PMID 39164512). Same-window (SW) columns are the present analysis restricted to the aligned post-approval period, 2022 Q2–2023 Q3 (1,003 mavacamten cases versus 1,004 in Yukselen et al.); the aligned period covers the post-approval quarters available in the present database and is therefore described as aligned rather than identical to the published calendar window (January 2022–September 2023). Full-window (FW) columns are the present 2022 Q2–2025 Q2 analysis (2,933 mavacamten cases) and are shown for completeness only; published Yukselen values are compared exclusively with same-window values, never with full-window values. aROR = exploratory adjusted reporting odds ratio (full-window models). ROR = reporting odds ratio; IC = information component; CI = confidence interval; PT = preferred term.
